# Molecular Species Identification with Rich Floristic Sampling: DNA Barcoding the Pteridophyte Flora of Japan

**DOI:** 10.1371/journal.pone.0015136

**Published:** 2010-12-08

**Authors:** Atsushi Ebihara, Joel H. Nitta, Motomi Ito

**Affiliations:** 1 Department of Botany, National Museum of Nature and Science, Tsukuba-shi, Ibaraki, Japan; 2 Department of Integrative Biology, University of California, Berkeley, California, United States of America; 3 Departments of System Sciences, Graduate School of Arts and Sciences, University of Tokyo, Meguro-ku, Tokyo, Japan; University of Sydney, Australia

## Abstract

**Background:**

DNA barcoding is expected to be an effective identification tool for organisms with heteromorphic generations such as pteridophytes, which possess a morphologically simple gametophyte generation. Although a reference data set including complete coverage of the target local flora/fauna is necessary for accurate identification, DNA barcode studies including such rich taxonomic sampling on a countrywide scale are lacking.

**Methodology/Principal Findings:**

The Japanese pteridophyte flora (733 taxa including subspecies and varieties) was used to test the utility of two plastid DNA barcode regions (*rbcL* and *trnH*-*psbA*) with the intention of developing an identification system for native gametophytes. DNA sequences were obtained from each of 689 (94.0%) taxa for *rbcL* and 617 (84.2%) taxa for *trnH-psbA*. Mean interspecific divergence values across all taxon pairs (K2P genetic distances) did not reveal a significant difference in rate between *trnH-psbA* and *rbcL*, but mean K2P distances of each genus showed significant heterogeneity according to systematic position. The minimum fail rate of taxon discrimination in an identification test using BLAST (12.52%) was obtained when *rbcL* and *trnH-psbA* were combined, and became lower in datasets excluding infraspecific taxa or apogamous taxa, or including sexual diploids only.

**Conclusions/Significance:**

This study demonstrates the overall effectiveness of DNA barcodes for species identification in the Japanese pteridophyte flora. Although this flora is characterized by a high occurrence of apogamous taxa that pose a serious challenge to identification using DNA barcodes, such taxa are limited to a small number of genera, and only minimally detract from the overall success rate. In the case that a query sequence is matched to a known apogamous genus, routine species identification may not be possible. Otherwise, DNA barcoding is a practical tool for identification of most Japanese pteridophytes, and is especially anticipated to be helpful for identification of non-hybridizing gametophytes.

## Introduction

Pteridophytes (ferns and lycophytes) are the only land plants possessing distinct, free-living sporophyte (2n) and gametophyte (1n) generations. As the site of fertilization and recruitment, the gametophyte is highly ecologically significant; however, previous taxonomic and ecological studies on pteridophytes have largely been confined to the sporophyte generation because of the difficulties associated with finding and identifying tiny (<1 cm), morphologically simple gametophytes [1]. The relatively few studies on pteridophyte gametophyte ecology have shown that in some lineages this generation occupies different (often broader) ecological niches than the sporophyte generation [2]–[5], and in extreme cases may persist without producing sporophytes at all as ‘independent gametophytes’ [6]. Pteridophyte gametophytes are likely to be extremely important in determining the distribution of sporophytes, the success of populations, and ultimately, the evolution of the fern and lycophyte lineages.

DNA barcoding [7] is a tool that can potentially be used to rapidly identify pteridophyte gametophytes to the species level [8]–[10], thus enabling detailed field studies linking the distribution and ecology of pteridophyte gametophytes and sporophytes. However, a standardized protocol for DNA barcoding in plants is still being developed, and pteridophytes as a group have not yet been the focus of a DNA barcode study. Previous plant DNA barcoding studies have typically taken one of three sampling approaches: broad studies including many species pairs across diverse genera to identify universal markers with high species resolution (e.g. [11]–[16]), floristic studies testing a few markers for all the plants in a given geographic area (e.g. [11], [17]–[19]), and densely sampled taxonomic studies testing the applicability of proposed markers on a chosen group (e.g. [20]–[25]). The current study combines floristic and taxonomic approaches, and tests the applicability of two proposed plastid barcode markers, *rbcL* and *trnH-psbA*, on the pteridophytes of Japan. Although the Consortium for the Barcode of Life (CBoL) proposed two DNA barcode regions for plants (*rbcL* and *matK*)[26], the current universal primer set for *matK* fails for most pteridophytes; thus, *trnH*-*psbA*, one of the regions designated as a supplementary locus by CBoL [26], was used instead.

In addition to the ecological insights that can be gained from species-level identification of the gametophyte generation, pteridophytes represent an important group to test methods of identification using DNA barcodes because of their high rate of polyploids [27]. Because the chloroplast has been shown to be maternally inherited in all pteridophytes studied so far (e.g. [28]–[30]), plastid markers alone cannot distinguish allopolyploid taxa that have recovered fertility by chromosome doubling after hybridization from sexual, non-hybrid taxa; however, most proposed plant barcode protocols do not include nuclear markers due to difficulty in sequencing and complex molecular evolution [31]. One of the goals of the current study is to determine the capabilities of a DNA barcode system using only plastid markers to distinguish species in a polyploid-rich taxonomic group such as pteridophytes.

Apogamous reproduction, the formation of a sporophyte from a gametophyte without fertilization, is not a rare reproductive mode in ferns [32]. Apogamous ‘species’ are often morphologically intermediate between two sexual species and/or other apogamous species, probably as a result of overcoming hybrid sterility by acquisition of apogamy [33]. Therefore, apogamous taxa are expected to pose similar problems to DNA barcoding as sexual allopolyploids.

For practical application of DNA barcoding, a complete set of reference data that includes all the taxa that may occur in the study area is necessary. To our knowledge, however, there is no example of a DNA barcode reference data set that covers a whole country for any taxonomic group above the family level. The Japanese pteridophyte flora is an ideal target for a DNA barcode model study with complete flora sampling because 1) the flora is well-known not only at species-level but also for ploidy and reproductive mode, 2) the number of native species (approximately 650, although this number varies depending on breadth of species concept [34], [35]) which represent 32 of all 40 extant pteridophyte families sensu Smith et al. 2006 [36], is relatively rich considering its land area (378,000 km^2^), and 3) since the country is completely bounded by the sea, it stands not only as a political region but also a biogeographically significant area (notwithstanding the low rate of endemism of Japanese pteridophytes compared to flowering plants; [37]). Most DNA barcoding studies have neglected infraspecific taxa, but our sampling includes subspecific and varietal ranks. Considering that rates of pteridophyte species diversity are frequently underestimated as demonstrated by the discovery of a number of cryptic species [38], present infraspecific taxa may represent independent units (biological species or different polyploids). Sampling for the current study is based on our updated checklist of Japanese pteridophytes reflecting the results of the latest research, which enumerates 733 taxa including subspecies, varieties and a few naturalized species (A. Ebihara, unpublished data). The Japanese pteridophyte flora is also characterized by the presence of a large number of putative interspecific hybrids; Nakaike (2004) listed more than 300 combinations [39]. However, putatively F1 sterile hybrid taxa are not included in the present sampling because of the difficulty in complete collection.

Candidate DNA barcode regions are usually tested with multiple individuals per taxon, even if this does not include all taxa from a target flora or taxonomic group. However, we concentrated the present sampling on high coverage for the flora because a sampling strategy that includes multiple collections of some taxa while leaving many completely unsampled makes little sense for the discussion of marker discrimination ability in a local context. On the other hand, the results obtained from analysis of a single sample per taxon with almost complete taxon coverage may demonstrate a “maximum success rate” of species identification which will never increase but will probably decrease by adding multiple samples per taxon.

## Results

### Success rates for PCR and sequencing

*rbcL* sequences were obtained from all 689 DNA samples, while *trnH*-*psbA* sequences could only be obtained from 617 of the 652 samples attempted (94.6%). Polymerase chain reaction (PCR) of *trnH*-*psbA* was almost always successful (failed in only four samples [0.6%] after multiple trials using various PCR conditions), but problems frequently occurred during sequencing due to polyG repeats. In Selaginellaceae, contamination by algal sequences was observed in a significant number of samples (7/16 samples for *trnH*-*psbA*); contaminated samples were removed from the analysis.

### Divergence values

**Figure 1:**
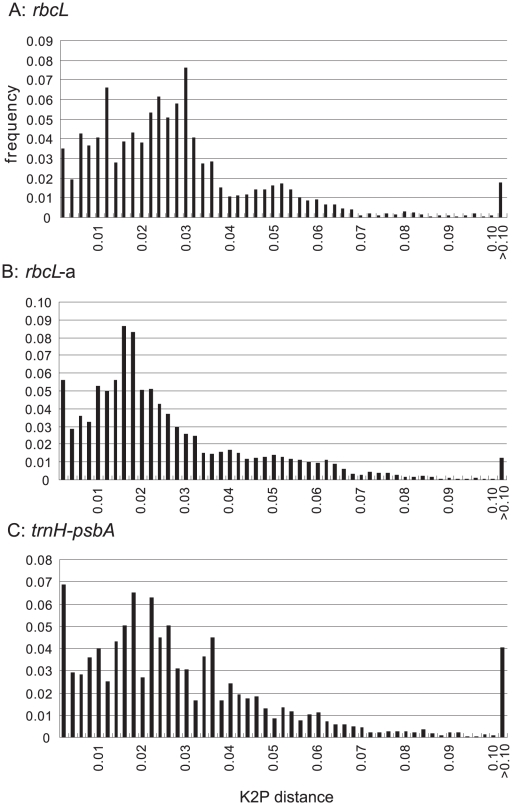
Distribution of interspecific K2P distances across all taxon pairs of Japanese pteridophytes. Infraspecific taxa are treated as distinct species. (A) *rbcL*, (B) *rbcL-*a, (C) *trnH*-*psbA*.

**Figure 2:**
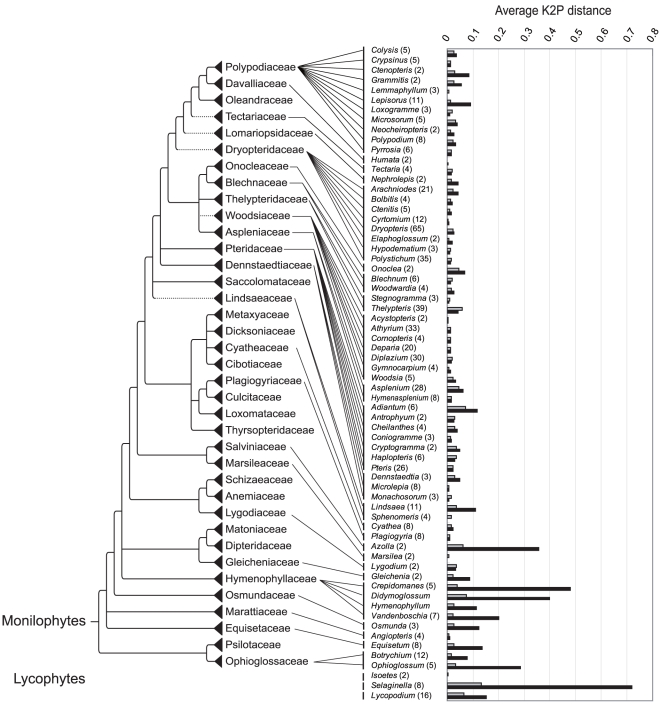
Average interspecific K2P distances for each genus containing multiple taxa (θ' values). Infraspecific taxa are treated as distinct species. θ'values are placed upon the latest family-level classification [36]. Gray: *rbcL*, black: *trnH*-*psbA*.

Figure 1 shows the distribution of interspecific K2P distances among all combinations of taxa whose sequences were successfully obtained for *trnH-psbA*, *rbcL*, and *rbcL-a* (the upper ca. 700 bp of *rbcL*). A comparison of average K2P distances within genera (θ' values) between *rbcL* and *trnH*-*psbA* is summarized in Figure 2. For the 66 genera that had θ' values for both *rbcL* and *trnH-psbA*, distances for *trnH*-*psbA* were significantly larger than those of *rbcL* (Wilcoxon test p = 1.98×10^−5^) and *rbcL*-a (p = 4.241×10^−6^).

### BLAST test

Summaries of species discrimination fail rates based on the three regions and their combinations are shown in Table 1 (among the all taxa) and Table 2 (within major families). The single region with the lowest fail rate is *rbcL* (22.08%). The combination of regions showing the lowest fail rate is *rbcL* + *trnH*-*psbA* (16.25%), and fail rates decreased if apogamous taxa or infraspecific taxa were excluded. Ploidies and reproductive modes are known for 78.3% of Japanese taxa (Supporting Information Table S1). When sampling was restricted to 254 taxa reported only as sexual diploids, fail rates decreased substantially: 7.91% in *rbcL* and 3.56% in *rbcL* + *trnH*-*psbA*.

**Table 1:** Results of BLAST species identification test.

	All taxa		Without apogamous taxa		Without infraspecific taxa		Sexual diploids only	
Region	N	Fail rate (%)	N	Fail rate (%)	N	Fail rate (%)	N	Fail rate (%)
*trnH*-*psbA*	598	34.95	527	30.36	559	29.87	225	16.89
*rbcL*	684	22.08	604	18.71	637	17.43	253	7.91
*rbcL-*a	684	29.09	604	25.17	637	25.27	253	10.67
*rbcL-*a + *trnH*-*psbA*	597	20.27	526	16.54	558	15.77	225	4.44
*rbcL* + *trnH*-*psbA*	597	16.25	526	12.36	558	12.37	225	3.56

**Table 2:** Results of BLAST species identification tests by major family (>20 taxa per family).

Family	Number of taxa listed in Table S1	Rate of apogamous taxa (%)	Rate of sexual diploids (%)	Fail rate (%)				
				*rbcL*	*rbcL*-a	*trnH-psbA*	*rbcL* + *trnH-psbA*	*rbcL*-a + *trnH-psbA*
Ophioglossaceae	21	0	38.1	28.57	42.86	27.78	11.11	11.11
Hymenophyllaceae	34	8.82	50.00	17.65	17.65	17.39	17.39	17.39
Pteridaceae	57	21.05	17.54	16.07	23.21	32.7	13.46	13.46
Aspleniaceae	42	9.52	14.29	4.76	14.29	11.43	0	5.71
Woodsiaceae	111	9.90	27.93	31.53	39.64	34.38	12.5	19.79
Thelypteridaceae	46	2.17	26.09	17.77	24.44	45.24	19.51	19.51
Dryopteridaceae	161	28.57	39.13	32.92	42.86	53.52	30.28	36.62
Polypodiaceae	60	1.67	45.00	8.33	8.33	24.14	3.45	3.45

## Discussion

The molecular divergence rate of *trnH*-*psbA* has been reported to be faster than *rbcL* in previous plant DNA barcode studies (e.g. [11]–[13], [16]). This trend was not observed in the distribution of interspecific K2P divergence values across all taxon combinations (Figure 1); however, comparison of mean divergence values within each genus (θ' values) by genus pair did statistically support an overall faster rate of divergence in *trnH-psbA* relative to *rbcL* (see Results). These seemingly conflicting results are best explained by a few species-rich genera (e.g. *Dryopteris*, *Thelypteris*, *Polystichum*) with low rates of divergence in *trnH-psbA* that skew the overall distribution of interspecific values. Furthermore, inspection of θ' values (Figure 2) reveals that the divergence rate of *trnH-psbA* seems to be correlated with phylogenetic position, apparently evolving more rapidly in early diverging groups and more slowly in recently diverging groups. Lycophytes and “basal” ferns showed relatively frequent sequence variation in *trnH-psbA* (e.g. θ' = 0.15 in *Lycopodium*, θ' = 0.48 in *Crepidomanes*) which is also reflected in Nitta's (2008) successful use of *trnH-psbA* for species identification in the early diverging fern family Hymenophyllaceae [40]. On the other hand, molecular divergence rates for *trnH-psbA* are much lower, and nearly equal to those of *rbcL* in eupolypods [41], the recently derived lineage including the bulk of extant fern diversity [42].

In the BLAST test, the minimum fail rate for discrimination (16.25%) was observed when *rbcL* and *trnH*-*psbA* were used in combination as a two-locus barcode. When *rbcL* was shortened to the upper 700 bp region (*rbcL-a*) as suggested in other DNA barcode studies [13], [14], [16], the fail rate increased to 20.27%. Species discrimination fail rates were also dependent on taxonomy: if we ignored infraspecific taxa, the fail rate decreased to 12.57%. Of course, we should bear in mind that these values are derived from a data set comprising only one sample per taxon, and that future addition of reference data will undoubtedly increase the fail rate. That said, it is an appropriate interpretation of the present results that species identification for Japanese pteridophyte flora succeeds in nearly 85% of the trials using a combination of full-length *rbcL* and *trnH-psbA*. *trnH*-*psbA* apparently works well as the second DNA barcode for pteridophytes instead of *matK*; nevertheless, *trnH*-*psbA* had fatal problems during sequencing caused by frequent polymers. In our case, only 75% of the samples could be successfully sequenced on the first trial. It is probably not worthwhile to invest in laborious sequencing protocols such as cloning [43] to obtain barcodes for a region that can only reduce the fail rate of the BLAST test by 7–15%, and in contrast, full-length *rbcL* as a single DNA barcode locus seems the more practical choice.

Since there are few examples of DNA barcoding studies with dense taxonomic sampling, it is difficult to compare our fail rates directly with those of previous studies. But our minimum fail rate values seem acceptable when compared with the results of recent DNA barcoding studies (Table 3, [13], [14], [44]. Debates over the relative performance of each candidate DNA barcode region aside, a certain degree of discrimination failure can be attributed to uncertainty of species circumscription. The Japanese pteridophyte flora is known to have a high occurrence of apogamous taxa, approximately 15% [45]. The apogamous habit shows a close relationship to phylogenetic position: 87% of apogamous species of Japan belong to only five genera in four families, namely *Cyrtomium* (Dryopteridaceae), *Dryopteris* (Dryopteridaceae), *Diplazium* (Woodsiaceae), *Pteris* (Pteridaceae) and *Hymenasplenum* (Aspleniaceae). Although the apogamous taxa of these genera have scientific names, many such ‘species’ in fact comprise morphologically ill-defined species complexes. The borders between multiple apogamous species are often ambiguous; in other words, the morphological variation within one apogamous species is often larger than closely related sexually reproducing species [46], [47]. These characteristics presumably reflect the origins of apogamous taxa – hybridization, recurrent origins and reticulate relationships [48]–[51] – and they lead to recognition of non-monophyletic species on the gene-tree. In fact, the observed fail rates of the BLAST test in families with a large proportion of apogamous taxa tended to be high (Table 2), and the BLAST test excluding apogamous taxa resulted in improved species resolution (Table 1). Discrimination failure attributed to non-monophyletic history will not be avoidable even if a codominant nuclear DNA marker is adopted as a DNA barcode. At present, there is no solution to the discrimination difficulty of apogamous taxa, and identification results based on molecular data need to be reviewed by specialists if the best-match taxon belongs to an apogamous-rich genus. Even if apogamous taxa are removed, allopolyploids, common in pteridophytes as noted above, are also expected to increase the fail rate. In the current study, the greatest reduction in fail rates was observed when sampling of the BLAST test was limited to only sexual, diploid taxa (Table 1).

**Table 3:** Comparison of minimum species discrimination fail rates in selected DNA barcoding studies.

Publication	Minimum fail rate	Barcode region	Sampling	Definition of ‘success’
Present study	16.25%	*rbcL* + *trnH*-*psbA*	597 individuals/597 taxa/60 genera of pteridophytes	Species matching 100% over the entire sequence length with their own reference sequence only
Kress and Erickson (2007) [13]	12.50%	*rbcL* + *trnH*-*psbA*	96 individuals/96 species [2 species per genus] of land plants	Proportion of genera in which species could be differentiated
Fazekas et al. (2008) [14]	36%	*rbcL* + *trnH*-*psbA*	251 individuals/92 species/32 genera of land plants	Inclusion of query sequence in any conspecific monophyletic group
Liu et al. (2010) [15]	11%	*rbcL*-a	100 individuals/58 taxa/53 genera of mosses	Inclusion of query sequence in any conspecific monophyletic group

Overall, DNA barcoding is a highly effective tool for identifying species among the Japanese pteridophyte flora, despite its high rate of apogamous taxa. Although the present study only includes a single sample per taxon, additional data will better characterize molecular variation and may reveal cryptic species, which are not uncommon in pteridophytes [52]. Furthermore, DNA barcoding using the present markers is expected to be particularly useful for identifying gametophytes, in which interspecific sterile F1 hybrids are usually not a concern. One exciting possible future result of our strategy of ‘complete regional taxonomic sampling’ may be the discovery of independently growing gametophytes that lack accompanying counterpart sporophytes within the same country.

## Materials and Methods

### Materials

DNA of all but 42 taxa listed in our checklist has been newly extracted for this study (DNA/sequences used in our previous studies were reused for some Hymenophylloid taxa) from their native localities or cultivated stocks in botanical gardens, reaching a final representation of 689/733 taxa ( = 94.0%) (see Supporting Information Table S1). Materials used were primarily sporophytic leaf tissue samples/cultivated stocks collected from localities in Japan, but conspecific material from outside the country was substituted for 17 taxa that are difficult to obtain from Japanese localities. All extracted DNA is deposited in the Center for Molecular Biodiversity Research, National Museum of Nature and Science.

### PCR and sequencing

DNA sequence data were obtained for two chloroplast regions, *rbcL* and *trnH*-*psbA*. For the *rbcL* gene, ca. 1400 bp was amplified using the primers F1 and 1379R [53], and sequencing reactions were performed using combinations of four primers: F1, 645F, 888R and 1379R [54], [55]. The *trnH*-*psbA* intergenic spacer was amplified using the primers psbA-F and trnH-R [56]; sequencing reactions were performed with PCR primers, but the following taxon-specific primers were also used for some samples: trnH-Rn1 (GGACGTGAACRAGATCTATC) mostly for Pteridaceae, psbA-Fn1 (CGTCTGGTTATGCAGCACAA) and trnH-Rn2 (CCTTGATCCACTTGGCTACG) both mainly for Dryopteridaceae.

### Measures of resolution

Three DNA barcode regions (*rbcL*-a [upper 700 bp of *rbcL*], full length *rbcL* [1205bp], and the *trnH*-*psbA* intergenic spacer [lengths ranged from 130 bp to 514 bp after deletion of primer regions]) were analyzed for their suitability as markers for species identification by characterizing interspecific divergence values and conducting a local BLAST search to test for rates of successful species identification. The *rbcL*-a region was tested for its utility as a DNA barcode marker by using trimmed *rbcL* sequences; hence, PCR and sequencing success rates of *rbcL*-a were not observed.

Intragenic, interspecific divergence values (K2P genetic distance) were calculated for each pair of species using PAUP* 4.0b10 [57] and averaged over all species pairs for each barcode region (*rbcL*, *rbcL*-a, and *trnH*-*psbA*) and combination of regions (*rbcL* + *trnH*-*psbA* and *rbcL*-a + *trnH*-*psbA*). Infraspecific taxa were treated as distinct species. *trnH*-*psbA* sequences could not be obtained for some taxa, so no statistical test was attempted to compare total average interspecific divergence values between barcode regions. Average interspecific divergence values were also calculated for each genus with more than one species (e.g. theta prime [θ'] values [58]) for each barcode region and combination of regions. The Wilcoxon Matched-Pairs Signed-Ranks Test was used to test for significant differences in θ' values between all pairs of genera for which both *rbcL* and *trnH*-*psbA* data were available.

A local BLAST search was used as a test of species identification ability. First, a reference library was constructed using the "makeblastDB" command in BLAST+ [59] for each region (*rbcL*, *rbcL*-a, and *trnH-psbA*) and combination of regions (*rbcL* + *trnH*-*psbA* and *rbcL*-a + *trnH*-*psbA*). The barcode sequence of each species was then queried against the reference library with the "blastn" command. Infraspecific taxa were treated as distinct species. Species matching 100% over the entire sequence length with their own reference sequence only were counted as successful identifications; those that also matched 100% over the entire sequence length with the reference sequence of one or more different species were counted as failures. Since a large portion of *trnH*-*psbA* sequences was missing data at one or both ends, the alignment was trimmed and 11 taxa with missing *trnH*-*psbA* data were removed from the BLAST analysis; the *rbcL* alignment did not require trimming, but three taxa with missing *rbcL* data were also removed (Supporting Information Table S2). Additional BLAST searches were conducted on the following datasets: apogamous (80 taxa) excluded, infraspecific (47 taxa) excluded, sexual diploids (253 taxa) only. Mean BLAST results were also calculated by family for each major family (>20 taxa per family).

## Supporting Information

Table S1 **List of plant material (voucher) and GenBank accession numbers.**
(DOC)

Table S2 **List of samples removed from the BLAST test.**
(DOC)
