# Supplementary material for: Molecular Species Identification with Rich Floristic Sampling: DNA Barcoding the Pteridophyte Flora of Japan
Source: PLoS One. 2010 Dec 8;5(12):e15136. doi: 10.1371/journal.pone.0015136 (PMC2999545; doi:10.1371/journal.pone.0015136)
Supplement: Table S1 — List of plant material (voucher) and GenBank accession numbers. (DOC) [file pone.0015136.s001.doc]

Table S1 List of plant material (voucher) and GenBank accession numbers.

|  | Species | Reproductive mode / Ploidy | Country | Prefecture | | Herbarium | Reg. No. | GenBank Accession |  | | | |  |
| --- | --- | --- | --- | --- | --- | --- | --- | --- | --- | --- | --- | --- | --- |
|  |  |  |  |  | |  |  | *rbcL* | *trnH-psbA* | | | |  |
| Lycopodiaceae | |  |  |  | |  |  |  |  | | | |  |
|  | *Lycopodium annotinum* L. | 1 | Japan | Nagano Pref. | | TNS | 765139 | AB574622 | AB575301 | | | |  |
|  | *Lycopodium carolinianum* L.  (= *Lycopodiella caroliniana* (L.) Pic.Serm.) | 2 | Japan | Shiga Pref. | | TNS | 743681 | AB574623 | N/A | | | |  |
|  | *Lycopodium casuarinoides* Spring | 1 | Japan | Kumamoto Pref. | | TNS | 762656 | AB574624 | AB575302 | | | |  |
|  | *Lycopodium cernuum* L.  (= *Lycopodiella cernua* (L.) Pic.Serm.) | 2 | Japan | Okinawa Pref. | | TNS | 759265 | AB574625 | N/A | | | |  |
|  | *Lycopodium clavatum* L. | 2 | Japan | Kagoshima Pref. | | TNS | 762554 | AB574626 | AB575303 | | | |  |
|  | *Lycopodium complanatum* L. | 1 | Japan | Akita Pref. | | TNS | 765154 | AB574627 | AB575304 | | | |  |
|  | *Lycopodium cryptomerinum* Maxim.  (= *Huperzia cryptomerina* (Maxim.) Dixit) | 2 | Japan | Kagoshima Pref. | | TNS | 763172 | AB574628 | AB575305 | | | |  |
|  | *Lycopodium fargesii* Herter  (= *Huperzia fargesii* (Herter) Holub) | 0 | Japan | Kagoshima Pref. | | FU | Yahara et al. Yaku-0398 | AB574629 | AB575306 | | | |  |
|  | *Lycopodium fordii* Baker  (= *Huperzia fordii* (Baker) Dixit) | 2 | Japan | Kagoshima Pref. | | TNS | 763058 | AB574630 | AB575307 | | | |  |
|  | *Lycopodium inundatum* L.  (= *Lycopodiella inundata* (L.) Holub) | 2 | Japan | Akita Pref. | | TNS | 765256 | AB574631 | AB575308 | | | |  |
|  | *Lycopodium miyoshianum* Makino  (= *Huperzia miyoshiana* (Makino) Ching) | 2 | Japan | Kagoshima Pref. | | TNS | 763253 | AB574632 | N/A | | | |  |
|  | *Lycopodium nikoense* (Franch. et Sav.) Franch. et Sav. | 1 | Japan | Shizuoka Pref. | | TNS | 766482 | AB574633 | AB575309 | | | |  |
|  | *Lycopodium obscurum* L. | 1 | Japan | Nagano Pref. | | TNS | 765137 | AB574634 | AB575310 | | | |  |
|  | *Lycopodium phlegmaria* L.  (= *Huperzia phlegmaria* (L.) Rothm.) | 2 | Japan | Kagoshima Pref. | | TNS | 763399 | AB574635 | AB575311 | | | |  |
|  | *Lycopodium selago* L.  (= *Huperzia selago* (L.) Bernh. ex Schrank et C.F.P.Mart.) | 2 | Japan | Yamagata Pref. | | TNS | 765813 | AB574636 | AB575312 | | | |  |
|  | *Lycopodium serratum* Thunb.  (=Huperzia serrata (Thunb.) Trevis.) | 2 | Japan | Kagoshima Pref. | | TNS | 763064 | AB574637 | AB575313 | | | |  |
|  | *Lycopodium sieboldii* Miq. var. *sieboldii* (=*Huperzia sieboldii* (Miq.) Holub) | 2 | Japan | Kagoshima Pref. | | TNS | 763308 | AB574638 | AB575314 | | | |  |
|  | *Lycopodium sieboldii* Miq. var. *christensenianum* (H.Christ et Herter) Tagawa  (=*Huperzia sieboldii* (Miq.) Holub var. *christenseniana* (H.Christ et Herter) Nakaike) | 0 | Japan | Kagoshima Pref. | | FU | Yahara et al. Yaku-0176 | AB574639 | AB575315 | | | |  |
|  | *Lycopodium somae* Hayata  (= *Huperzia somae* (Hayata) Ching) | 0 | Taiwan |  | | TNS | 776522 | AB574640 | AB575316 | | | |  |
| Selaginellaceae | |  |  |  | |  |  |  |  | | | |  |
|  | *Selaginella biformis* A.Braun ex Kuhn | 1 | Japan | Okinawa Pref. | | TNS | 777848 | AB574641 | AB575317 | | | |  |
|  | *Selaginella boninensis* Baker | 1 | Japan | Tokyo Pref. | | TNS | 766618 | AB574642 | N/A | | | |  |
|  | *Selaginella doederleinii* Hieron. | 1 | Japan | Kagoshima Pref. | | TNS | 763070 | AB574643 | AB575318 | | | |  |
|  | *Selaginella helvetica* (L.) Spring | 1 | Japan | Akita Pref. | | TNS | 765114 | AB574644 | AB575319 | | | |  |
|  | *Selaginella heterostachys* Baker | 2 | Japan | Mie Pref. | | TNS | 769193 | AB574645 | AB575320 | | | |  |
|  | *Selaginella involvens* (Sw.) Spring | 1 | Japan | Kagoshima Pref. | | TNS | 762549 | AB574646 | N/A | | | |  |
|  | *Selaginella limbata* Alston | 1 | Japan | Kagoshima Pref. | | TNS | 764147 | AB574647 | AB575321 | | | |  |
|  | *Selaginella lutchuensis* Koidz. | 1 | Japan | Okinawa Pref. | | TNS | 759343 | AB574648 | N/A | | | |  |
|  | *Selaginella nipponica* Franch. et Sav. | 1 | Japan | Tokyo Pref. | | TNS | 738139 | AB574649 | N/A | | | |  |
|  | *Selaginella remotifolia* Spring | 1 | Japan | Shizuoka Pref. | | TNS | 765133 | AB574650 | AB575322 | | | |  |
|  | *Selaginella selaginoides* (L.) P.Beauv. ex Schrank et C.F.P.Mart. | 1 | Japan | Nagano Pref. | | TNS | 766485 | AB574651 | N/A | | | |  |
|  | *Selaginella shakotanensis* (Franch. ex Takeda) Miyabe et Kudô | 1 | Japan | Nagano Pref. | | TNS | 776371 | AB574652 | N/A | | | |  |
|  | *Selaginella sibirica* (Milde) Hieron. | 0 | Japan | Hokkaido Pref. | | TNS | 743691 | AB574653 | N/A | | | |  |
|  | *Selaginella tamamontana* Seriz. | 1 | Japan | Mie Pref. | | TNS | 769195 | AB574654 | AB575323 | | | |  |
|  | *Selaginella tamariscina* (P.Beauv.) Spring | 1 | Japan | Okinawa Pref. | | TNS | 759348 | AB574655 | N/A | | | |  |
|  | *Selaginella uncinata* (Desv.) Spring | 1 | Japan | Tokyo Pref. | | TNS | 9508552 | AB574656 | AB575324 | | | |  |
| Isoetaceae | |  |  |  | |  |  |  |  | | | |  |
|  | *Isoetes asiatica* (Makino) Makino | 1 | Japan | Fukushima Pref. | | TNS | 1107828 | AB574657 | N/A | | | |  |
|  | *Isoetes japonica* A.Braun | 2 | Japan | Ibaraki Pref. | | TNS | 763864 | AB574658 | AB575325 | | | |  |
|  | *Isoetes pseudojaponica* M.Takamiya, Mitsu.Watan. et K.Ono | 2 | Japan | Okayama Pref. | | TNS | 763994 | AB574659 | AB575326 | | | |  |
|  | *Isoetes sinensis* T.C.Palmer | 2 | Japan | Nagasaki Pref. | | TNS | 743727 | AB574660 | N/A | | | |  |
| Ophioglossaceae | |  |  |  | |  |  |  |  | | | |  |
|  | *Botrychium atrovirens* (Sahashi) M.Kato  (= *Sceptridium atrovirens* Sahashi) | 2 | Japan | Tokushima Pref. | | TNS | 771508 | AB574661 | AB575327 | | | |  |
|  | *Botrychium formosanum* Tagawa  (= *Sceptridium formosanum* (Tagawa) Holub) | 2 | Japan | Okinawa Pref. | | TNS | 759323 | AB574662 | AB575328 | | | |  |
|  | *Botrychium japonicum* (Prantl) Underw.  (= *Sceptridium japonicum* (Prantl) Lyon) | 2 | Japan | Chiba Pref. | | TNS | 1107868 | AB574663 | AB575329 | | | |  |
|  | *Botrychium lunaria* (L.) Sw. | 1 | Japan | Saitama Pref. | | TNS | 776399 | AB574664 | AB575330 | | | |  |
|  | *Botrychium microphyllum* (Sahashi) K.Iwats.  (= *Sceptridium microphyllum* Sahashi) | 1 | Japan | Hokkaido Pref. | | TNS | 1107870 | AB574665 | AB575331 | | | |  |
|  | *Botrychium multifidum* (S.G.Gmel.) Rupr. var. *multifidum*  *(= Sceptridium multifidum* (S.G.Gmel.) M.Nishida) | 1 | Japan | Hokkaido Pref. | | TNS | 1107871 | AB574666 | AB575332 | | | |  |
|  | *Botrychium multifidum* (S.G.Gmel.) Rupr. var. *robustum* (Rupr. ex Milde) C.Chr.  (= *Sceptridium multifidum* (S.G.Gmel.) M.Nishida var. *robustum* (Rupr. ex Milde) M.Nishida) | 1 | Japan | Fukushima Pref. | | TNS | 1107867 | AB574667 | AB575333 | | | |  |
|  | *Botrychium nipponicum* Makino  (= *Sceptridium nipponicum* (Makino) Holub) | 1 | Japan | Ibaraki Pref. | | TNS | 1107878 | AB574668 | AB575334 | | | |  |
|  | *Botrychium strictum* Underw. | 1 | Japan | Kanagawa Pref. | | TNS | 765136 | AB574669 | AB575335 | | | |  |
|  | *Botrychium ternatum* (Thunb.) Sw. var. *ternatum*  (= *Sceptridium ternatum* (Thunb.) Lyon) | 1 | Japan | Miyagi Pref. | | TNS | 1107869 | AB574670 | AB575336 | | | |  |
|  | *Botrychium ternatum* (Thunb.) Sw. var. *pseudoternatum* (Sahashi) M.Kato  (= *Sceptridium ternatum* (Thunb.) Lyon var. *pseudoternatum* Sahashi) | 0 | Japan | Chiba Pref. | | CBM | Kimura s.n. | AB574671 | N/A | | | |  |
|  | *Botrychium triangularifolium* (Sahashi) M.Kato  (= *Sceptridium triangularifolium* Sahashi) | 1 | Japan | Tokyo Pref. | | TNS | 771440 | AB574672 | AB575337 | | | |  |
|  | *Botrychium virginianum* (L.) Sw. | 2 | Japan | Shizuoka Pref. | | TNS | 764332 | AB574673 | AB575338 | | | |  |
|  | *Helminthostachys zeylanica* (L.) Hook. | 0 | Japan | Okinawa Pref. | | TNS | 759310 | AB574674 | AB575339 | | | |  |
|  | *Ophioglossum namegatae* M.Nishida et Kurita | 2 | Japan | Ibaraki Pref. | | TNS | 764351 | AB574675 | AB575340 | | | |  |
|  | *Ophioglossum parvum* M.Nishida et Kurita | 2 | Japan | Shizuoka Pref. | | TNS | 1108350 | AB574676 | N/A | | | |  |
|  | *Ophioglossum pendulum* L.  (= *Ophioderma pendulum* (L.) C.Presl) | 0 | Japan | Okinawa Pref. | | TNS | 759347 | AB574677 | AB575341 | | | |  |
|  | *Ophioglossum petiolatum* Hook. | 2 | Japan | Kagoshima Pref. | | TNS | 764216 | AB574678 | AB575342 | | | |  |
|  | *Ophioglossum thermale* Kom. var. *thermale* | 2 | Japan | Okinawa Pref. | | TNS | 764007 | AB574679 | AB575343 | | | |  |
|  | *Ophioglossum thermale* Kom. var. *nipponicum* (Miyabe et Kudô) M.Nishida | 2 | Japan | Tokyo Pref. | | TNS | 1108349 | AB574680 | N/A | | | |  |
|  | *Ophioglossum vulgatum* L. | 2 | Japan | Chiba Pref. | | TNS | 1108351 | AB574681 | AB575344 | | | |  |
| Psilotaceae | |  |  |  | |  |  |  |  | | | |  |
|  | *Psilotum nudum* (L.) P.Beauv. | 2 | Japan | Kagoshima Pref. | | TNS | 763307 | AB574682 | AB575345 | | | |  |
| Equisetaceae | |  |  |  | |  |  |  |  | | | |  |
|  | *Equisetum arvense* L. | 1 | Japan | Fukuoka Pref. | | TNS | 763618 | AB574683 | AB575346 | | | |  |
|  | *Equisetum fluviatile* L. | 0 | Japan | Aomori Pref. | | TNS | 776384 | AB574684 | AB575347 | | | |  |
|  | *Equisetum hyemale* L. | 0 | Japan | Iwate Pref. | | TNS | 765859 | AB574685 | AB575348 | | | |  |
|  | *Equisetum palustre* L. | 0 | Japan | Chiba Pref. | | TNS | 776981 | AB574686 | AB575349 | | | |  |
|  | *Equisetum pratense* Ehrh. | 0 | Japan | Hokkaido Pref. | | TNS | 1107843 | AB574687 | N/A | | | |  |
|  | *Equisetum ramosissimum* Desf. | 0 | Japan | Ibaraki Pref. | | TNS | 763922 | AB574688 | AB575350 | | | |  |
|  | *Equisetum scirpoides* Michx. | 0 | Japan | Hokkaido Pref. | | TNS | 768168 | AB574689 | AB575351 | | | |  |
|  | *Equisetum sylvaticum* L. | 0 | USA | Alaska | | TNS | 776974 | AB574690 | AB575352 | | | |  |
|  | *Equisetum variegatum* Schleich. ex F.Weber et D.Mohr | 0 | Japan | Gunma Pref. | | TNS | 768164 | AB574691 | AB575353 | | | |  |
| Marattiaceae | |  |  |  | |  |  |  |  | | | |  |
|  | *Angiopteris boninensis* Hieron. | 1 | Japan | Tokyo Pref. | | TNS | 766499 | AB574692 | AB575354 | | | |  |
|  | *Angiopteris fokiensis* Hieron. | 0 | Japan | Kagoshima Pref. | | TNS | 771442 | AB574693 | AB575355 | | | |  |
|  | *Angiopteris lygodiifolia* Rosenst. | 1 | Japan | Kagoshima Pref. | | TNS | 763473 | AB574694 | AB575356 | | | |  |
|  | *Angiopteris palmiformis* (Cav.) C.Chr. | 1 | Taiwan |  | | TNS | 763875 | AB574695 | AB575357 | | | |  |
| Osmundaceae | |  |  |  | |  |  |  |  | | | |  |
|  | *Osmunda banksiifolia* (C.Presl) Kuhn | 1 | Japan | Okinawa Pref. | | TNS | 759261 | AB574696 | AB575358 | | | |  |
|  | *Osmunda claytoniana* L. | 1 | Japan | Nagano Pref. | | TNS | 764334 | AB574697 | AB575359 | | | |  |
|  | *Osmunda japonica* Thunb. | 1 | Japan | Miyazaki Pref. | | TNS | 9531687 | AB574698 | N/A | | | |  |
|  | *Osmunda lancea* Thunb. | 1 | Japan | Shizuoka Pref. | | TNS | 764347 | AB574699 | AB575360 | | | |  |
|  | *Osmundastrum cinnamomeum* (L.) C.Presl var. *fokiense* (Copel.) Tagawa  (= *Osmunda cinnamomea* L. var. *fokiensis* Copel.) | 1 | Japan | Akita Pref. | | TNS | 765171 | AB574700 | N/A | | | |  |
| Hymenophyllaceae | |  |  |  | |  |  |  |  | | | |  |
|  | *Abrodictyum boninense* Tagawa et K.Iwats.  (= *Cephalomanes boninense* (Tagawa et K.Iwats.) K.Iwats.) | 1 | Japan | Tokyo Pref. | | TNS | 1108352 | AB257472 | N/A | | | |  |
|  | *Abrodictyum obscurum* (Blume) Ebihara et K.Iwats.  (= *Selenodesmium obscurum* (Blume) Copel.) | 1 | Japan | Okinawa Pref. | | TNS | 759307 | AB574701 | AB575361 | | | |  |
|  | *Callistopteris apiifolia* (C.Presl) Copel. | 1 | Japan | Kagoshima Pref. | | TNS | 736797 | AB574702 | AB575362 | | | |  |
|  | *Cephalomanes javanicum* (Blume) Bosch var. *asplenioides* (C.Chr.) K.Iwats. | 1 | Japan | Okinawa Pref. | | TNS | 759314 | AB574703 | AB575363 | | | |  |
|  | *Crepidomanes acuto-obtusum* (Hayata) K.Iwats. | 1 | Japan | Tokyo Pref. | | TNS | 1108431 | AB574704 | AB575364 | | | |  |
|  | *Crepidomanes bipunctatum* (Poir.) Copel. | 1 | Japan | Okinawa Pref. | | TNS | 759345 | AB574705 | N/A | | | |  |
|  | *Crepidomanes humile* (G.Forst.) Bosch  (= *Reediella humilis* (G.Forst.) Pich.Ser.) | 0 | Japan | Okinawa Pref. | | TNS | 759340 | AB574706 | N/A | | | |  |
|  | *Crepidomanes kurzii* (Bedd.) Tagawa et K.Iwats. | 0 | Japan | Okinawa Pref. | | TNS | 766608 | AB574707 | N/A | | | |  |
|  | *Crepidomanes latealatum* (Bosch) Copel. | 1 | Japan | Tokyo Pref. | | TNS | 1108353 | AB574708 | N/A | | | |  |
|  | *Crepidomanes latemarginale* (D.C.Eaton) Copel. | 3 | Taiwan |  | | TNS | 1108354 | AB257468 | AB575365 | | | |  |
|  | *Crepidomanes makinoi* (C.Chr.) Copel. | 0 | Japan | Kochi Pref. | | TNS | 776936 | AB574709 | AB575366 | | | |  |
|  | *Crepidomanes minutum* (Blume) K.Iwats. | 3 | Japan | Kagoshima Pref. | | TNS | 762567 | AB574710 | AB575367 | | | |  |
|  | *Crepidomanes schmidtianum* (Zenker ex Taschner) K.Iwats. | 3 | Japan | Nagano Pref. | | TNS | 776489 | AB574711 | N/A | | | |  |
|  | *Crepidomanes thysanostomum* (Makino) Ebihara et K.Iwats.  (= *Nesopteris thysanostoma* (Makino) Copel.) | 2 | Japan | Okinawa Pref. | | TNS | 759288 | AB574712 | AB575368 | | | |  |
|  | *Didymoglossum bimarginatum* (Bosch) Ebihara et K.Iwats.  (= *Microgonium bimarginatum* (Bosch) Bosch) | 0 | Taiwan |  | | TNS | 777865 | AB574713 | AB575369 | | | |  |
|  | *Didymoglossum motleyi* (Bosch) Ebihara et K.Iwats.  (= *Microgonium motleyi* Bosch) | 0 | Japan | Okinawa Pref. | | TNS | 759342 | AB574714 | AB575370 | | | |  |
|  | *Didymoglossum tahitense* (Nadeaud) Ebihara et K.Iwats.  (= *Microgonium tahitense* (Nadeaud) Tindale) | 0 | Japan | Okinawa Pref. | | TNS | 759341 | AB574715 | AB575371 | | | |  |
|  | *Hymenophyllum badium* Hook. et Grev.  (= *Mecodium badium* (Hook. et Grev.) Copel.) | 0 | Japan | Mie Pref. | | TNS | 1108355 | AB191440 | N/A | | | |  |
|  | *Hymenophyllum barbatum* (Bosch) Baker | 1 | Japan | Kagoshima Pref. | | TNS | 762568 | AB574716 | AB575372 | | | |  |
|  | *Hymenophyllum coreanum* Nakai  (= *Mecodium coreanum* (Nakai) Seriz.) | 0 | Japan | Aomori Pref. | | TNS | 1108356 | AB574717 | N/A | | | |  |
|  | *Hymenophyllum denticulatum* Sw.  (= *Meringium denticulatum* (Sw.) Copel.) | 0 | Japan | Wakayama Pref. | | TNS | 1108357 | AB574718 | N/A | | | |  |
|  | *Hymenophyllum flexile* Makino  (= *Mecodium flexile* (Makino) Copel.) | 0 | Japan | Kagoshima Pref. | | TNS | 764259 | AB574719 | AB575373 | | | |  |
|  | *Hymenophyllum mikawanum* (Seriz.) Seriz.  (= *Mecodium mikawanum* Seriz.) | 0 | Japan | Aichi Pref. | | TNS | 738136 | AB574720 | N/A | | | |  |
|  | *Hymenophyllum oligosorum* Makino  (= *Mecodium oligosorum* (Makino) H.Itô) | 1 | Japan | Miyazaki Pref. | | TNS | 762777 | AB574721 | AB575374 | | | |  |
|  | *Hymenophyllum polyanthos* (Sw.) Sw.  (= *Mecodium polyanthos* (Sw.) Copel.) | 1 | Japan | Kagoshima Pref. | | TNS | 773469 | AB574722 | AB575375 | | | |  |
|  | *Hymenophyllum riukiuense* H.Christ  (= *Mecodium riukiuense* (H.Christ) Copel.) | 0 | Japan | Okinawa Pref. | | TNS | 1108358 | AB574723 | N/A | | | |  |
|  | *Hymenophyllum wrightii* Bosch  (= *Mecodium wrightii* (Bosch) Copel.) | 1 | Japan | Akita Pref. | | TNS | 765790 | AB574724 | AB575376 | | | |  |
|  | *Vandenboschia auriculata* (Blume) Copel. | 1 | Japan | Okinawa Pref. | | TNS | 759317 | AB574725 | AB575377 | | | |  |
|  | *Vandenboschia birmanica* (Bedd.) Ching | 1 | Japan | Kagoshima Pref. | | TNS | 778952 | AB196367 | AB575378 | | | |  |
|  | *Vandenboschia kalamocarpa* (Hayata) Ebihara | 1 | Japan | Tokyo Pref. | | TNS | 778666 | AB196364 | AB575379 | | | |  |
|  | *Vandenboschia liukiuensis* (Y.Yabe) Tagawa | 1 | Japan | Kagoshima Pref. | | TNS | 778913 | AB196369 | AB575380 | | | |  |
|  | *Vandenboschia maxima* (Blume) Copel. | 0 | Japan | Okinawa Pref. | | TNS | 1108359 | AB574726 | AB575381 | | | |  |
|  | *Vandenboschia nipponica* (Nakai) Ebihara | 1 | Japan | Aomori Pref. | | TNS | 778936 | AB196366 | AB575382 | | | |  |
|  | *Vandenboschia subclathrata* K.Iwats. | 1 | Japan | Okinawa Pref. | | TNS | 778354 | AB196364 | AB575383 | | | |  |
| Gleicheniaceae | |  |  |  | |  |  |  |  | | | |  |
|  | *Dicranopteris linearis* (Burm.f.) Underw. | 1 | Japan | Okinawa Pref. | | TNS | 759368 | AB574727 | AB575384 | | | |  |
|  | *Gleichenia japonica* Spreng. | 1 | Japan | Kagoshima Pref. | | TNS | 763375 | AB574728 | AB575385 | | | |  |
|  | *Gleichenia laevissima* H.Christ | 1 | Japan | Kagoshima Pref. | | TNS | 762581 | AB574729 | AB575386 | | | |  |
| Dipteridaceae | |  |  |  | |  |  |  |  | | | |  |
|  | *Cheiropleuria integrifolia* (D.C.Eaton ex Hook.) M.Kato, Y.Yatabe, Sahashi e N.Murak. t | 2 | Japan | Kagoshima Pref. | | TNS | 763051 | AB574730 | AB575387 | | | |  |
|  | *Dipteris conjugata* Reinw. | 1 | Japan | Okinawa Pref. | | TNS | 759252 | AB574731 | AB575388 | | | |  |
| Lygodiaceae | |  |  |  | |  |  |  |  | | | |  |
|  | *Lygodium japonicum* (Thunb.) Sw. | 2 | Japan | Miyazaki Pref. | | TNS | 762774 | AB574732 | AB575389 | | | |  |
|  | *Lygodium microphyllum* (Cav.) R.Br. | 1 | Japan | Okinawa Pref. | | TNS | 759309 | AB574733 | AB575390 | | | |  |
| Schizaeaceae | |  |  |  | |  |  |  |  | | | |  |
|  | *Schizaea dichotoma* (L.) Sm. | 0 | Japan | Okinawa Pref. | | TNS | 9533740 | AB574734 | N/A | | | |  |
|  | *Schizaea digitata* (L.) Sw. | 2 | Japan | Tokyo Pref. | | TNS | 766616 | AB574735 | AB575391 | | | |  |
| Marisileaceae | |  |  |  | |  |  |  |  | | | |  |
|  | *Marsilea crenata* C.Presl | 1 | Japan | Fukuoka Pref. | | TNS | 763648 | AB574736 | AB575392 | | | |  |
|  | *Marsilea quadrifolia* L. | 1 | Japan | Chiba Pref. | | TNS | 776982 | AB574737 | AB575393 | | | |  |
| Salviniaceae | |  |  |  | |  |  |  |  | | | |  |
|  | *Azolla imbricata* (Roxb. ex Griff.) Nakai | 1 | Japan | Okinawa Pref. | | TNS | 769192 | AB574738 | AB575394 | | | |  |
|  | *Azolla japonica* (Franch. et Sav.) Franch. et Sav. ex Nakai | 1 | Japan | Hyogo Pref. | | TNS | 776374 | AB574739 | AB575395 | | | |  |
|  | *Salvinia natans* (L.) All. | 1 | Japan | Chiba Pref. | | TNS | 777856 | AB574740 | AB575396 | | | |  |
| Plagiogyriaceae | |  |  |  | |  |  |  |  | | | |  |
|  | *Plagiogyria adnata* (Blume) Bedd. var. *adnata* | 2 | Japan | Kagoshima Pref. | | TNS | 763110 | AB574741 | AB575397 | | | |  |
|  | *Plagiogyria adnata* (Blume) Bedd. var. *yakushimensis* (Kansei Sato) Tagawa | 2 | Japan | Kagoshima Pref. | | TNS | 764126 | AB574742 | AB575398 | | | |  |
|  | *Plagiogyria euphlebia* (Kunze) Mett. | 2 | Japan | Kagoshima Pref. | | TNS | 763111 | AB574743 | AB575399 | | | |  |
|  | *Plagiogyria japonica* Nakai | 2 | Japan | Kagoshima Pref. | | TNS | 763065 | AB574744 | AB575400 | | | |  |
|  | *Plagiogyria koidzumii* Tagawa | 0 | Japan | Okinawa Pref. | | TNS | 9533792 | AB574745 | AB575401 | | | |  |
|  | *Plagiogyria matsumureana* Makino | 1 | Japan | Hokkaido Pref. | | TNS | 765673 | AB574746 | AB575402 | | | |  |
|  | *Plagiogyria stenoptera* (Hance) Diels | 0 | Japan | Kagoshima Pref. | | TNS | 763183 | AB574747 | AB575403 | | | |  |
|  | *Plagiogyria yakumonticola* Nakaike | 0 | Japan | Kagoshima Pref. | | TNS | 763938 | AB574748 | AB575404 | | | |  |
| Cibotiaceae | |  |  |  | |  |  |  |  | | | |  |
|  | *Cibotium barometz* (L.) J.Sm. | 1 | Japan | Okinawa Pref. | | TNS | 759294 | AB574749 | AB575405 | | | |  |
| Cyatheaceae | |  |  |  | |  |  |  |  | | | |  |
|  | *Cyathea hancockii* Copel. | 2 | Japan | Kagoshima Pref. | | TNS | 764241 | AB574750 | AB575406 | | | |  |
|  | *Cyathea lepifera* (J.Sm. ex Hook.) Copel. | 1 | Japan | Okinawa Pref. | | TNS | 774833 | AB574751 | AB575407 | | | |  |
|  | *Cyathea mertensiana* (Kunze) Copel. | 1 | Japan | Tokyo Pref. | | TNS | 768155 | AB574752 | AB575408 | | | |  |
|  | *Cyathea metteniana* (Hance) C.Chr. et Tardieu | 2 | Japan | Kagoshima Pref. | | TNS | 763048 | AB574753 | AB575409 | | | |  |
|  | *Cyathea ogurae* (Hayata) Domin | 1 | Japan | Tokyo Pref. | | TNS | 763924 | AB574754 | AB575410 | | | |  |
|  | *Cyathea podophylla* (Hook.) Copel. | 1 | Japan | Okinawa Pref. | | TNS | 759302 | AB574755 | AB575411 | | | |  |
|  | *Cyathea spinulosa* Wall. ex Hook. | 1 | Taiwan |  | | TNS | 763916 | AB574756 | AB575412 | | | |  |
|  | *Cyathea tuyamae* H.Ohba | 0 | Japan | Tokyo Pref. | | MAK | Takayama 07062131 | AB574757 | AB575413 | | | |  |
| Lindsaeaceae | |  |  |  | |  |  |  |  | | | |  |
|  | *Lindsaea cambodgensis* H.Christ | 0 | Japan | Kagoshima Pref. | | TNS | 777864 | AB574758 | AB575414 | | | |  |
|  | *Lindsaea chienii* Ching | 1 | Japan | Kagoshima Pref. | | TNS | 763146 | AB574759 | AB575415 | | | |  |
|  | *Lindsaea ensifolia* Sw. | 0 | Japan | Okinawa Pref. | | TNS | 743779 | AB574760 | AB575416 | | | |  |
|  | *Lindsaea heterophylla* Dryand. | 0 | Japan | Kagoshima Pref. | | TNS | 764258 | AB574761 | AB575417 | | | |  |
|  | *Lindsaea javanensis* Blume | 0 | Japan | Kagoshima Pref. | | TNS | 763479 | AB574762 | AB575418 | | | |  |
|  | *Lindsaea kawabatae* Sa.Kurata | 0 | Japan | Kagoshima Pref. | | TNS | 773438 | AB574763 | AB575419 | | | |  |
|  | *Lindsaea lucida* Blume | 1 | Japan | Okinawa Pref. | | TNS | 759336 | AB574764 | AB575420 | | | |  |
|  | *Lindsaea merrillii* Copel. subsp. *yaeyamensis* (Tagawa) K.U.Kramer | 1 | Japan | Okinawa Pref. | | TNS | 759283 | AB574765 | AB575421 | | | |  |
|  | *Lindsaea odorata* Roxb. var. *odorata* | 2 | Japan | Kagoshima Pref. | | TNS | 763121 | AB574766 | AB575422 | | | |  |
|  | *Lindsaea odorata* Roxb. var. *japonica* (Baker) K.U.Kramer | 2 | Japan | Okinawa Pref. | | TNS | 759306 | AB574767 | AB575423 | | | |  |
|  | *Lindsaea orbiculata* (Lam.) Mett. ex Kuhn var. *orbiculata* |  | Japan | Okinawa Pref. | | TNS | 759263 | AB574768 | AB575424 | | | |  |
|  | *Lindsaea orbiculata* (Lam.) Mett. ex Kuhn var. *commixta* (Tagawa) K.U.Kramer | 2 | Japan | Okinawa Pref. | | TNS | 759248 | AB574769 | AB575425 | | | |  |
|  | *Lindsaea repanda* Kunze | 1 | Japan | Tokyo Pref. | | TNS | 743688 | AB574770 | N/A | | | |  |
|  | *Lindsaea simulans* Ching | 0 | Japan | Kochi Pref. | | TNS | 766619 | AB574771 | AB575426 | | | |  |
|  | *Sphenomeris biflora* (Kaulf.) Tagawa  (= *Odontosoria biflora* (Kaulf.) C.Chr.) | 1 | Japan | Okinawa Pref. | | TNS | 759259 | AB574772 | AB575427 | | | |  |
|  | *Sphenomeris chinensis* (L.) Maxon  (= *Odontosoria chinensis* (L.) J.Sm.) | 2 | Japan | Kagoshima Pref. | | TNS | 763305 | AB574773 | AB575428 | | | |  |
|  | *Sphenomeris gracilis* (Tagawa) Sa.Kurata | 1 | Japan | Okinawa Pref. | | TNS | 759282 | AB574774 | AB575429 | | | |  |
|  | *Sphenomeris minutula* Sa.Kurata | 1 | Japan | Kagoshima Pref. | | TNS | 764192 | AB574775 | AB575430 | | | |  |
|  | *Tapeinidium pinnatum* (Cav.) C.Chr. | 2 | Japan | Okinawa Pref. | | TNS | 759253 | AB574776 | AB575431 | | | |  |
| Dennstaedtiaceae | |  |  |  | |  |  |  |  | | | |  |
|  | *Dennstaedtia hirsuta* (Sw.) Mett. | 1 | Japan | Ibaraki Pref. | | TNS | 763870 | AB574777 | AB575432 | | | |  |
|  | *Dennstaedtia scabra* (Wall. ex Hook.) T.Moore | 2 | Japan | Kagoshima Pref. | | TNS | 763127 | AB574778 | AB575433 | | | |  |
|  | *Dennstaedtia wilfordii* (T.Moore) H.Christ ex C.Chr. | 1 | Japan | Tokyo Pref. | | TNS | 763999 | AB574779 | AB575434 | | | |  |
|  | *Histiopteris incisa* (Thunb.) J. Sm. | 2 | Japan | Wakayama Pref. | | TNS | 766402 | AB574780 | AB575435 | | | |  |
|  | *Hypolepis punctata* (Thunb.) Mett. ex Kuhn | 2 | Japan | Mie Pref. | | TNS | 766424 | AB574781 | AB575436 | | | |  |
|  | *Microlepia hookeriana* (Wall. ex Hook.) C.Presl | 0 | Japan | Okinawa Pref. | | TNS | 764009 | AB574782 | AB575437 | | | |  |
|  | *Microlepia izupeninsulae* Sa.Kurata | 2 | Japan | Shizuoka Pref. | | TNS | 766448 | AB574783 | AB575438 | | | |  |
|  | *Microlepia marginata* (Panzer ex Houtt.) C.Chr. | 2 | Japan | Kagoshima Pref. | | TNS | 763417 | AB574784 | AB575439 | | | |  |
|  | *Microlepia obtusiloba* Hayata | 2 | Japan | Kagoshima Pref. | | TNS | 763302 | AB574785 | AB575440 | | | |  |
|  | *Microlepia pseudostrigosa* Makino | 2 | Japan | Shizuoka Pref. | | TNS | 766447 | AB574786 | AB575441 | | | |  |
|  | *Microlepia speluncae* (L.) T.Moore | 2 | Japan | Okinawa Pref. | | TNS | 764027 | AB574787 | AB575442 | | | |  |
|  | *Microlepia strigosa* (Thunb.) C.Presl | 1 | Japan | Kagoshima Pref. | | TNS | 764170 | AB574788 | AB575443 | | | |  |
|  | *Microlepia substrigosa* Tagawa | 2 | Japan | Kochi Pref. | | TNS | 771555 | AB574789 | AB575444 | | | |  |
|  | *Monachosorum arakii* Tagawa | 2 | Japan | Kumamoto Pref. | | TNS | 762680 | AB574790 | AB575445 | | | |  |
|  | *Monachosorum flagellare* (Maxim. ex Makino) Hayata | 2 | Japan | Saitama Pref. | | TNS | 763927 | AB574791 | AB575446 | | | |  |
|  | *Monachosorum maximowiczii* (Baker) Hayata | 1 | Japan | Saitama Pref. | | TNS | 763926 | AB574792 | AB575447 | | | |  |
|  | *Pteridium aquilinum* (L.) Kuhn var. *latiusculum* (Desv.) Undrew. ex Hell. | 1 | Japan | Kagoshima Pref. | | TNS | 763350 | AB574793 | AB575448 | | | |  |
| Pteridaceae | |  |  |  | |  |  |  |  | | | |  |
|  | *Acrostichum aureum* L. | 2 | Japan | Okinawa Pref. | | TNS | 764385 | AB574794 | AB575449 | | | |  |
|  | *Adiantum capillus-junonis* Rupr. | 1 | Japan | Oita Pref. | | TNS | 743693 | AB574795 | N/A | | | |  |
|  | *Adiantum capillus-veneris* L. | 1 | Japan | Oita Pref. | | TNS | 774832 | AB574796 | AB575450 | | | |  |
|  | *Adiantum diaphanum* Blume | 0 | Taiwan |  | | TNS | 777847 | AB574797 | AB575451 | | | |  |
|  | *Adiantum edgeworthii* Hook. | 1 | Japan | Oita Pref. | | TNS | 743692 | AB574798 | N/A | | | |  |
|  | *Adiantum flabellulatum* L. | 0 | Japan | Okinawa Pref. | | TNS | 764008 | AB574799 | AB575452 | | | |  |
|  | *Adiantum monochlamys* D.C.Eaton | 2 | Japan | Tokyo Pref. | | TNS | 764001 | AB574800 | AB575453 | | | |  |
|  | *Adiantum ogasawarense* Tagawa | 0 | Japan | Tokyo Pref. | | TNS | 774843 | AB574801 | AB575454 | | | |  |
|  | *Adiantum pedatum* L. | 1 | Japan | Shizuoka Pref. | | TNS | 764338 | AB574802 | AB575455 | | | |  |
|  | *Antrophyum formosanum* Hieron. | 0 | Japan | Okinawa Pref. | | TNS | 776987 | AB574803 | AB575456 | | | |  |
|  | *Antrophyum obovatum* Baker | 0 | Japan | Kochi Pref. | | TNS | 766621 | AB574804 | AB575457 | | | |  |
|  | *Ceratopteris thalictroides* (L.) Brongn. | 2 | Japan | Ibaraki Pref. | | TNS | 764378 | AB574805 | AB575458 | | | |  |
|  | *Cheilanthes argentea* (S.G.Gmel.) Kunze | 2 | Japan | Iwate Pref. | | TNS | 765868 | AB574806 | AB575459 | | | |  |
|  | *Cheilanthes brandtii* Franch. et Sav. | 1 | Japan | Saitama Pref. | | TNS | 765116 | AB574807 | AB575460 | | | |  |
|  | *Cheilanthes chusana* Hook. | 3 | Japan | Kochi Pref. | | TNS | 764374 | AB574808 | AB575461 | | | |  |
|  | *Cheilanthes krameri* Franch. et Sav. | 0 | Japan | Tokyo Pref. | | TNS | 766462 | AB574809 | AB575462 | | | |  |
|  | *Coniogramme gracilis* Ogata | 1 | Japan | Kagoshima Pref. | | TNS | 766455 | AB574810 | AB575463 | | | |  |
|  | *Coniogramme intermedia* Hieron. var. *intermedia* | 2 | Japan | Kagoshima Pref. | | TNS | 762609 | AB574811 | AB575464 | | | |  |
|  | *Coniogramme intermedia* Hieron. var. *villosa* Ching | 0 | Japan | Saitama Pref. | | TNS | 766454 | AB574812 | N/A | | | |  |
|  | *Coniogramme japonica* (Thunb.) Diels | 2 | Japan | Shizuoka Pref. | | TNS | 766505 | AB574813 | AB575465 | | | |  |
|  | *Cryptogramma crispa* (L.) R.Br. ex Richards. | 0 | Japan | Akita Pref. | | TNS | 776983 | AB574814 | AB575466 | | | |  |
|  | *Cryptogramma stelleri* (S.G.Gmel.) Prantl | 1 | Japan | Nagano Pref. | | TNS | 766486 | AB574815 | AB575467 | | | |  |
|  | *Haplopteris ensiformis* (Sw.) E.H.Crane  (= *Vittaria ensiformis* Sw.) | 2 | Japan | Okinawa Pref. | | TNS | 759357 | AB574816 | AB575468 | | | |  |
|  | *Haplopteris flexuosa* (Fée) E.H.Crane  (= *Vittaria flexuosa* Fée) | 0 | Japan | Kagoshima Pref. | | TNS | 763122 | AB574817 | AB575469 | | | |  |
|  | *Haplopteris forrestiana* (Ching) E.H.Crane  (= *Vittaria forrestiana* Ching) | 0 | Japan | Kagoshima Pref. | | TNS | 736790 | AB574818 | AB575470 | | | |  |
|  | *Haplopteris fudzinoi* (Makino) E.H.Crane  (= *Vittaria fudzinoi* Makino) | 0 | Japan | Nara Pref. | | TNS | 766642 | AB574819 | AB575471 | | | |  |
|  | *Haplopteris zosterifolia* (Willd.) E.H.Crane  (= *Vittaria zosterifolia* Willd.) | 0 | Japan | Okinawa Pref. | | TNS | 759322 | AB574820 | AB575472 | | | |  |
|  | *Onychium japonicum* (Thunb.) Kunze | 2 | Japan | Kagoshima Pref. | | TNS | 762536 | AB574821 | AB575473 | | | |  |
|  | *Pityrogramma calomelanos* (L.) Link [introduced] | 0 | Taiwan |  | | TNS | 761580 | AB574822 | AB575474 | | | |  |
|  | *Pteris boninensis* H.Ohba | 1 | Japan | Tokyo Pref. | | TNS | 766502 | AB574823 | AB575475 | | | |  |
|  | *Pteris cadieri* H.Christ | 3 | Japan | Okinawa Pref. | | TNS | 759320 | AB574824 | AB575476 | | | |  |
|  | *Pteris cretica* L. | 3 | Japan | Kagoshima Pref. | | TNS | 762611 | AB574825 | AB575477 | | | |  |
|  | *Pteris deltodon* Baker | 2 | Japan | Kumamoto Pref. | | TNS | 762674 | AB574826 | AB575478 | | | |  |
|  | *Pteris dispar* Kunze | 2 | Japan | Miyazaki Pref. | | TNS | 762701 | AB574827 | AB575479 | | | |  |
|  | *Pteris ensiformis* Burm.f. | 2 | Japan | Kagoshima Pref. | | TNS | 764151 | AB574828 | AB575480 | | | |  |
|  | *Pteris excelsa* Gaudich. | 3 | Japan | Kagoshima Pref. | | TNS | 762557 | AB574829 | AB575481 | | | |  |
|  | *Pteris fauriei* Hieron. | 3 | Japan | Kagoshima Pref. | | TNS | 763416 | AB574830 | AB575482 | | | |  |
|  | *Pteris formosana* Baker | 0 | Japan | Kagoshima Pref. | | TNS | 764239 | AB574831 | N/A | | | |  |
|  | *Pteris grevilleana* Wall. ex J.Agardh | 3 | Japan | Kagoshima Pref. | | TNS | 736792 | AB574832 | AB575483 | | | |  |
|  | *Pteris kawabatae* Sa.Kurata | 0 | Japan | Kagoshima Pref. | | TNS | 736791 | AB574833 | AB575484 | | | |  |
|  | *Pteris kidoi* Sa.Kurata | 1 | Japan | Kumamoto Pref. | | TNS | 763915 | AB574834 | AB575485 | | | |  |
|  | *Pteris kiuschiuensis* Hieron. | 3 | Japan | Kagoshima Pref. | | TNS | 763326 | AB574835 | AB575486 | | | |  |
|  | *Pteris laurisilvicola* Sa.Kurata | 3 | Japan | Wakayama Pref. | | TNS | 766415 | AB574836 | AB575487 | | | |  |
|  | *Pteris multifida* Poir. | 2 | Japan | Kumamoto Pref. | | TNS | 762654 | AB574837 | AB575488 | | | |  |
|  | *Pteris nakasimae* Tagawa | 0 | Japan | Kagoshima Pref. | | TNS | 771437 | AB574838 | AB575489 | | | |  |
|  | *Pteris natiensis* Tagawa | 3 | Japan | Miyazaki Pref. | | TNS | 763883 | AB574839 | AB575490 | | | |  |
|  | *Pteris nipponica* W.C.Shieh | 3 | Japan | Miyazaki Pref. | | TNS | 762737 | AB574840 | AB575491 | | | |  |
|  | *Pteris oshimensis* Hieron. | 3 | Japan | Wakayama Pref. | | TNS | 766469 | AB574841 | AB575492 | | | |  |
|  | *Pteris ryukyuensis* Tagawa | 2 | Japan | Kagoshima Pref. | | TNS | 764234 | AB574842 | AB575493 | | | |  |
|  | *Pteris semipinnata* L. | 2 | Japan | Kagoshima Pref. | | TNS | 763419 | AB574843 | AB575494 | | | |  |
|  | *Pteris setulosocostulata* Hayata | 3 | Japan | Kagoshima Pref. | | TNS | 763292 | AB574844 | AB575495 | | | |  |
|  | *Pteris tokioi* Masam. | 2 | Japan | Miyazaki Pref. | | TNS | 763889 | AB574845 | AB575496 | | | |  |
|  | *Pteris vittata* L. | 2 | Japan | Kagoshima Pref. | | TNS | 764121 | AB574846 | AB575497 | | | |  |
|  | *Pteris wallichiana* J.Agardh | 1 | Japan | Shizuoka Pref. | | TNS | 765134 | AB574847 | AB575498 | | | |  |
|  | *Pteris yakuinsularis* Sa.Kurata | 0 | Japan | Miyazaki Pref. | | TNS | 762741 | AB574848 | AB575499 | | | |  |
|  | *Pteris yamatensis* (Tagawa) Tagawa | 2 | Japan | Nara Pref. | | TNS | 774837 | AB574849 | AB575500 | | | |  |
|  | *Vittaria* (*Haplopteris*) *ogasawarensis* Kodama | 0 | Japan | Tokyo Pref. | | TNS | 774844 | AB574850 | AB575501 | | | |  |
| Aspleniaceae | |  |  |  | |  |  |  |  | | | |  |
|  | *Asplenium antiquum* Makino | 2 | Japan | Kagoshima Pref. | | TNS | 763113 | AB574851 | AB575502 | | | |  |
|  | *Asplenium boreale* (Ohwi ex Sa.Kurata) Nakaike | 0 | Japan | Wakayama Pref. | | TNS | 766401 | AB574853 | AB575504 | | | |  |
|  | *Asplenium capillipes* Makino | 1 | Japan | Kochi Pref. | | TNS | 766625 | AB574854 | AB575505 | | | |  |
|  | *Asplenium coenobiale* Hance | 0 | Japan | Kochi Pref. | | TNS | 763925 | AB574855 | AB575506 | | | |  |
|  | *Asplenium ensiforme* Wall. ex Hook. et Grev. | 0 | Japan | Miyazaki Pref. | | TNS | 763995 | AB574856 | AB575507 | | | |  |
|  | *Asplenium griffithianum* Hook. | 0 | Japan | Kagoshima Pref. | | FU | Fujita et al. Yaku-1204 | AB574857 | AB575508 | | | |  |
|  | *Asplenium incisum* Thunb. | 1 | Japan | Kumamoto Pref. | | TNS | 762637 | AB574858 | AB575509 | | | |  |
|  | *Asplenium laserpitiifolium* Lam. | 0 | Japan | Okinawa Pref. | | TNS | 743674 | AB574859 | N/A | | | |  |
|  | *Asplenium loriceum* H.Christ ex C.Chr. | 0 | Taiwan |  | | TNS | 763862 | AB574860 | AB575510 | | | |  |
|  | *Asplenium micantifrons* (Tuyama) Tuyama ex H.Ohba | 0 | Japan | Tokyo Pref. | | MAK | Takayama 07062176 | AB574861 | AB575511 | | | |  |
|  | *Asplenium nidus* L. | 2 | Japan | Okinawa Pref. | | TNS | 1107865 | AB574862 | N/A | | | |  |
|  | *Asplenium normale* D.Don | 0 | Japan | Kagoshima Pref. | | TNS | 763101 | AB574863 | AB575512 | | | |  |
|  | *Asplenium oligophlebium* Baker var. *oligophlebium* | 1 | Japan | Kagoshima Pref. | | TNS | 763209 | AB574864 | AB575513 | | | |  |
|  | *Asplenium oligophlebium* Baker var. *iezimaense* (Tagawa) Tagawa | 0 | Japan | Okinawa Pref. | | TNS | 743684 | AB574865 | N/A | | | |  |
|  | *Asplenium pekinense* Hance | 2 | Japan | Miyazaki Pref. | | TNS | 762770 | AB574866 | AB575514 | | | |  |
|  | *Asplenium polyodon* G.Forst. | 0 | Japan | Tokyo Pref. | | TNS | 743676 | AB574867 | N/A | | | |  |
|  | *Asplenium prolongatum* Hook. | 0 | Japan | Kumamoto Pref. | | TNS | 764386 | AB574868 | AB575515 | | | |  |
|  | *Asplenium pseudowilfordii* Tagawa | 2 | Japan | Saitama Pref. | | TNS | 776375 | AB574869 | AB575516 | | | |  |
|  | *Asplenium ritoense* Hayata | 2 | Japan | Kagoshima Pref. | | TNS | 762535 | AB574870 | AB575517 | | | |  |
|  | *Asplenium ruprechtii* Sa.Kurata | 1 | Japan | Kumamoto Pref. | | TNS | 762675 | AB574871 | AB575518 | | | |  |
|  | *Asplenium ruta-muraria* L. | 0 | Japan | Iwate Pref. | | TNS | 765881 | AB574872 | AB575519 | | | |  |
|  | *Asplenium sarelii* Hook. | 2 | Japan | Mie Pref. | | TNS | 769194 | AB574873 | AB575520 | | | |  |
|  | *Asplenium scolopendrium* L.  (= *Phyllitis scolopendrium* (L.) Newm.) | 2 | Japan | Yamagata Pref. | | TNS | 765242 | AB574874 | AB575521 | | | |  |
|  | *Asplenium setoi* N.Murak. et Seriz. | 2 | Japan | Okinawa Pref. | | TNS | 736798 | AB574875 | N/A | | | |  |
|  | *Asplenium shimurae* (H.Itô) Nakaike | 0 | Japan | Wakayama Pref. | | TNS | 766418 | AB574876 | AB575522 | | | |  |
|  | *Asplenium tenerum* G.Forst. | 0 | Japan | Tokyo Pref. | | TNS | 774845 | AB574877 | AB575523 | | | |  |
|  | *Asplenium tenuicaule* Hayata | 1 | Japan | Yamagata Pref. | | TNS | 765223 | AB574878 | AB575524 | | | |  |
|  | *Asplenium trichomanes* L. | 2 | Japan | Nara Pref. | | TNS | 766640 | AB574879 | AB575525 | | | |  |
|  | *Asplenium trigonopterum* Kunze | 3 | Japan | Tokyo Pref. | | TNS | 763893 | AB574880 | AB575526 | | | |  |
|  | *Asplenium tripteropus* Nakai | 2 | Japan | Miyazaki Pref. | | TNS | 762700 | AB574881 | AB575527 | | | |  |
|  | *Asplenium viride* Huds. | 1 | Japan | Saitama Pref. | | TNS | 776412 | AB574882 | AB575528 | | | |  |
|  | *Asplenium wilfordii* Mett. ex Kuhn | 2 | Japan | Kagoshima Pref. | | TNS | 763478 | AB574883 | AB575529 | | | |  |
|  | *Asplenium wrightii* D.C.Eaton ex Hook. | 2 | Japan | Miyazaki Pref. | | TNS | 762719 | AB574884 | AB575530 | | | |  |
|  | *Asplenium yoshinagae* Makino | 2 | Japan | Kumamoto Pref. | | TNS | 762691 | AB574885 | N/A | | | |  |
|  | *Hymenasplenium apogamum* (N.Murak. et S.-I.Hatan.) Nakaike  (= *Asplenium apogamum* N.Murak. et S.-I.Hatan.) | 3 | Japan | Okinawa Pref. | | TNS | 1107829 | AB574852 | AB575503 | | | |  |
|  | *Hymenasplenium cardiophyllum* (Hance) Nakaike  (= *Asplenium cardiophyllum* (Hance) Baker) | 2 | Japan | Okinawa Pref. | | TNS | 774848 | AB574886 | AB575531 | | | |  |
|  | *Hymenasplenium cheilosorum* (Kunze ex Mett.) Tagawa  (= *Asplenium cheilosorum* Kunze ex Mett.) | 3 | Japan | Kagoshima Pref. | | TNS | 763055 | AB574887 | AB575532 | | | |  |
|  | *Hymenasplenium excisum* (C.Presl) Hatus.  (= *Asplenium excisum* C.Presl) |  | Japan | Kagoshima Pref. | | TNS | 764169 | AB574888 | AB575533 | | | |  |
|  | *Hymenasplenium hondoense* (N.Murak. et S.-I.Hatan.) Nakaike  (= *Asplenium hondoense* N.Murak. et S.-I.Hatan.) | 3 | Japan | Tokyo Pref. | | TNS | 766457 | AB574889 | AB575534 | | | |  |
|  | *Hymenasplenium mitanii* Nakaike, nom. nud. | 0 | Japan | Kagoshima Pref. | | TNS | 736793 | AB574890 | AB575535 | | | |  |
|  | *Hymenasplenium murakami-hatanakae* Nakaike  (= *Asplenium cataractarum* Rosenst.) | 2 | Japan | Mie Pref. | | TNS | 763888 | AB574891 | AB575536 | | | |  |
|  | *Hymenasplenium obliquissimum* (Hayata) Sugim.  (= *Asplenium obliquissimum* (Hayata) Sugim. et Sa.Kurata) | 2 | Japan | Kagoshima Pref. | | TNS | 763123 | AB574892 | AB575537 | | | |  |
| Woodsiaceae | |  |  |  | |  |  |  |  | | | |  |
|  | *Acystopteris japonica* (Luerss.) Nakai | 1 | Japan | Tokyo Pref. | | TNS | 763998 | AB574893 | AB575538 | | | |  |
|  | *Acystopteris tenuisecta* (Blume) Tagawa | 0 | Japan | Kagoshima Pref. | | TNS | 763154 | AB574894 | AB575539 | | | |  |
|  | *Athyrium arisanense* (Hayata) Tagawa | 2 | Taiwan |  | | TNS | 762059 | AB574895 | AB575540 | | | |  |
|  | *Athyrium atkinsonii* Bedd. | 0 | Japan | Nagano Pref. | | TNS | 766635 | AB574896 | AB575541 | | | |  |
|  | *Athyrium brevifrons* Nakai ex Tagawa | 1 | Japan | Hokkaido Pref. | | TNS | 765672 | AB574897 | N/A | | | |  |
|  | *Athyrium clivicola* Tagawa | 2 | Japan | Akita Pref. | | TNS | 765182 | AB574898 | AB575542 | | | |  |
|  | *Athyrium deltoidofrons* Makino | 2 | Japan | Iwate Pref. | | TNS | 765857 | AB574899 | AB575543 | | | |  |
|  | *Athyrium distentifolium* Tausch ex Opiz | 1 | Japan | Akita Pref. | | TNS | 765803 | AB574900 | AB575544 | | | |  |
|  | *Athyrium eremicola* Oka et Sa.Kurata | 2 | Japan | Shimane Pref. | | TNS | 768163 | AB574901 | AB575545 | | | |  |
|  | *Athyrium frangulum* Tagawa | 1 | Japan | Mie Pref. | | TNS | 766433 | AB574902 | AB575546 | | | |  |
|  | *Athyrium iseanum* Rosenst. var. *iseanum* | 2 | Japan | Shizuoka Pref. | | TNS | 763880 | AB574903 | AB575547 | | | |  |
|  | *Athyrium iseanum* Rosenst. var. *angustisectum* Tagawa | 2 | Japan | Mie Pref. | | TNS | 763882 | AB574904 | AB575548 | | | |  |
|  | *Athyrium kirisimaense* Tagawa | 1 | Japan | Kagoshima Pref. | | TNS | 763280 | AB574905 | AB575549 | | | |  |
|  | *Athyrium kuratae* Seriz. | 2 | Japan | Kumamoto Pref. | | TNS | 762678 | AB574906 | AB575550 | | | |  |
|  | *Athyrium masamunei* Seriz. | 0 | Japan | Kagoshima Pref. | | FU | Yahara et al. Yaku-0523 | AB574907 | AB575551 | | | |  |
|  | *Athyrium melanolepis* (Franch. et Sav.) H.Christ | 1 | Japan | Akita Pref. | | TNS | 765161 | AB574908 | AB575552 | | | |  |
|  | *Athyrium nakanoi* Makino | 2 | Japan | Kagoshima Pref. | | TNS | 763067 | AB574909 | AB575553 | | | |  |
|  | *Athyrium neglectum* Seriz. subsp. *neglectum* | 0 | Japan | Akita Pref. | | TNS | 765794 | AB574910 | AB575554 | | | |  |
|  | *Athyrium neglectum* Seriz. subsp. *australe* Seriz. | 0 | Japan | Oita Pref. | | TNS | 776387 | AB574911 | AB575555 | | | |  |
|  | *Athyrium nikkoense* Makino | 1 | Japan | Saitama Pref. | | TNS | 776396 | AB574912 | AB575556 | | | |  |
|  | *Athyrium niponicum* (Mett.) Hance | 1 | Japan | Tokyo Pref. | | TNS | 776369 | AB574913 | AB575557 | | | |  |
|  | *Athyrium oblitescens* Sa.Kurata | 2 | Japan | Mie Pref. | | TNS | 766435 | AB574914 | AB575558 | | | |  |
|  | *Athyrium otophorum* (Miq.) Koidz. var. *otophorum* | 2 | Japan | Shizuoka Pref. | | TNS | 765119 | AB574915 | AB575559 | | | |  |
|  | *Athyrium otophorum* (Miq.) Koidz. var. *okanum* Sa.Kurata | 2 | Japan | Yamaguchi Pref. | | TNS | 743749 | AB574916 | N/A | | | |  |
|  | *Athyrium palustre* Seriz. | 0 | Japan | Kagoshima Pref. | | TNS | 763278 | AB574917 | AB575560 | | | |  |
|  | *Athyrium pinetorum* Tagawa | 1 | Japan | Nagano Pref. | | TNS | 766465 | AB574918 | AB575561 | | | |  |
|  | *Athyrium reflexipinnum* Hayata | 2 | Japan | Kagoshima Pref. | | TNS | 763929 | AB574919 | AB575562 | | | |  |
|  | *Athyrium rupestre* Kodama | 1 | Japan | Akita Pref. | | TNS | 765177 | AB574920 | AB575563 | | | |  |
|  | *Athyrium setuligerum* Sa.Kurata | 2 | Japan | Kumamoto Pref. | | TNS | 776386 | AB574921 | AB575564 | | | |  |
|  | *Athyrium sheareri* (Baker) Ching  (= *Anisocampium sheareri* (Baker) Ching) | 2 | Japan | Shimane Pref. | | TNS | 765141 | AB574922 | AB575565 | | | |  |
|  | *Athyrium silvicola* Tagawa | 0 | Taiwan |  | | TNS | 776662 | AB574923 | AB575566 | | | |  |
|  | *Athyrium spinulosum* (Maxim.) Milde | 1 | Japan | Saitama Pref. | | TNS | 776413 | AB574924 | AB575567 | | | |  |
|  | *Athyrium strigillosum* (Lowe) T.Moore ex Salomon | 2 | Japan | Kumamoto Pref. | | TNS | 743752 | AB574925 | N/A | | | |  |
|  | *Athyrium subrigescens* (Hayata) Hayata ex H.Itô | 2 | Japan | Kagoshima Pref. | | TNS | 763186 | AB574926 | AB575568 | | | |  |
|  | *Athyrium tashiroi* Tagawa | 1 | Japan | Nagasaki Pref. | | TNS | 743726 | AB574927 | N/A | | | |  |
|  | *Athyrium tozanense* (Hayata) Hayata | 2 | Japan | Kagoshima Pref. | | TNS | 763296 | AB574928 | AB575569 | | | |  |
|  | *Athyrium vidalii* (Franch. et Sav.) Nakai | 2 | Japan | Kagoshima Pref. | | TNS | 763224 | AB574929 | N/A | | | |  |
|  | *Athyrium viridescentipes* Sa.Kurata | 2 | Japan | Kumamoto Pref. | | TNS | 777858 | AB574930 | AB575570 | | | |  |
|  | *Athyrium wardii* (Hook.) Makino var. *wardii* | 2 | Japan | Akita Pref. | | TNS | 743724 | AB574931 | N/A | | | |  |
|  | *Athyrium wardii* (Hook.) Makino var. *inadae* Tagawa | 0 | Japan | Mie Pref. | | TNS | 766644 | AB574932 | AB575571 | | | |  |
|  | *Athyrium yokoscense* (Franch. et Sav.) H.Christ | 1 | Japan | Wakayama Pref. | | TNS | 766400 | AB574933 | AB575572 | | | |  |
|  | *Cornopteris banajaoensis* (C.Chr.) K.Iwats. et Price | 1 | Taiwan |  | | TNS | 761633 | AB574934 | AB575573 | | | |  |
|  | *Cornopteris crenulatoserrulata* (Makino) Nakai  (= *Athyrium crenulatoserrulatum* Makino) | 1 | Japan | Nagano Pref. | | TNS | 766634 | AB574935 | AB575574 | | | |  |
|  | *Cornopteris decurrenti-alata* (Hook.) Nakai  (= *Athyrium decurrenti-alatum* (Hook.) Copel.) | 2 | Japan | Saitama Pref. | | TNS | 765127 | AB574936 | AB575575 | | | |  |
|  | *Cornopteris opaca* (D.Don) Tagawa f. *glabrescens* Sa.Kurata  (= *Athyrium opacum* (D.Don) Copel. f. *glabrescens* (Sa.Kurata) Seriz.) | 2 | Taiwan |  | | TNS | 763909 | AB574937 | AB575576 | | | |  |
|  | *Cystopteris fragilis* (L.) Bernh. | 2 | Japan | Nagano Pref. | | TNS | 766489 | AB574938 | N/A | | | |  |
|  | *Cystopteris sudetica* A.Braun et Milde | 2 | Japan | Nagano Pref. | | TNS | 766629 | AB574939 | AB575577 | | | |  |
|  | *Deparia bonincola* (Nakai) M.Kato  (= *Lunathyrium bonincola* (Nakai) H.Ohba) | 1 | Japan | Tokyo Pref. | | TNS | 774841 | AB574940 | AB575578 | | | |  |
|  | *Deparia conilii* (Franch. et Sav.) M.Kato  (= *Lunathyrium conilii* (Franch. et Sav.) Sa.Kurata) | 2 | Japan | Tokyo Pref. | | TNS | 768165 | AB574941 | AB575579 | | | |  |
|  | *Deparia coreana* (H.Christ) M.Kato  (= *Lunathyrium coreanum* (H.Christ) Ching) | 0 | Japan | Aomori Pref. | | TNS | 776382 | AB574942 | AB575580 | | | |  |
|  | *Deparia dimorphophylla* (Koidz.) M.Kato  (= *Lunathyrium dimorphophyllum* (Koidz.) Sa.Kurata) | 2 | Japan | Kagoshima Pref. | | TNS | 764256 | AB574943 | AB575581 | | | |  |
|  | *Deparia formosana* (Rosenst.) R.Sano  (= *Dictyodroma formosanum* (Rosenst.) Ching) | 1 | Japan | Kagoshima Pref. | | TNS | 776380 | AB574944 | AB575582 | | | |  |
|  | *Deparia japonica* (Thunb.) M.Kato  (= *Lunathyrium japonicum* (Thunb.) Sa.Kurata) | 2 | Japan | Ibaraki Pref. | | TNS | 763869 | AB574945 | AB575583 | | | |  |
|  | *Deparia kiusiana* (Koidz.) M.Kato | 0 | Japan | Nara Pref. | | TNS | 764364 | AB574946 | AB575584 | | | |  |
|  | *Deparia lancea* (Thunb.) Fraser-Jenk.  (= *Diplazium subsinuatum* (Wall. ex Hook. et Grev.) Tagawa) | 2 | Japan | Fukuoka Pref. | | TNS | 763644 | AB574947 | AB575585 | | | |  |
|  | *Deparia minamitanii* Seriz. | 0 | Japan | Miyazaki Pref. | | TNS | 774852 | AB574948 | AB575586 | | | |  |
|  | *Deparia okuboana* (Makino) M.Kato  (= *Dryoathyrium okuboanum* (Makino) Ching) | 3 | Japan | Shizuoka Pref. | | TNS | 764345 | AB574949 | AB575587 | | | |  |
|  | *Deparia otomasui* (Sa.Kurata) Seriz.  (= *Lunathyrium otomasui* Sa.Kurata) | 1 | Japan | Kumamoto Pref. | | TNS | 764339 | AB574950 | AB575588 | | | |  |
|  | *Deparia petersenii* (Kunze) M.Kato  (= *Lunathyrium petersenii* (Kuntze) H.Ohba) | 2 | Japan | Kagoshima Pref. | | TNS | 763129 | AB574951 | AB575589 | | | |  |
|  | *Deparia pseudoconilii* (Seriz.) Seriz. var. *pseudoconilii*  (= *Lunathyrium pseudoconilii* Seriz.) | 0 | Japan | Okinawa Pref. | | TNS | 764016 | AB574952 | AB575590 | | | |  |
|  | *Deparia pseudoconilii* (Seriz.) Seriz. var. *subdeltoidofrons* (Seriz.) Seriz.  (= *Lunathyrium pseudoconilii* Seriz. var. *subdeltoidofrons* Seriz.) | 0 | Japan | Tokyo Pref. | | TNS | 776379 | AB574953 | AB575591 | | | |  |
|  | *Deparia pterorachis* (H.Christ) M.Kato  (= *Lunathyrium pterorachis* (H.Christ) Sa.Kurata) | 1 | Japan | Nagano Pref. | | TNS | 766637 | AB574954 | AB575592 | | | |  |
|  | *Deparia pycnosora* (H.Christ) M.Kato var. *pycnosora*  (= *Lunathyrium pycnosorum* (H.Christ) Koidz.) | 2 | Japan | Hokkaido Pref. | | TNS | 765674 | AB574955 | AB575593 | | | |  |
|  | *Deparia pycnosora* (H.Christ) M.Kato var. *albosquamata* M.Kato | 0 | Japan | Tokyo Pref. | | TNS | 763932 | AB574956 | AB575594 | | | |  |
|  | *Deparia pycnosora* (H.Christ) M.Kato var. *mucilagina* M.Kato | 0 | Japan | Saitama Pref. | | TNS | 763886 | AB574957 | AB575595 | | | |  |
|  | *Deparia unifurcata* (Baker) M.Kato  (= *Dryoathyrium unifurcatum* (Baker) Ching) | 3 | Japan | Nara Pref. | | TNS | 766643 | AB574958 | AB575596 | | | |  |
|  | *Deparia viridifrons* (Makino) M.Kato  (= *Dryoathyrium viridifrons* (Makino) Ching) | 1 | Japan | Nara Pref. | | TNS | 766472 | AB574959 | AB575597 | | | |  |
|  | *Diplaziopsis cavaleriana* (H.Christ) C.Chr.  (= *Diplazium cavalerianum* (H.Christ) M.Kato) | 2 | Japan | Nara Pref. | | TNS | 764367 | AB574960 | AB575598 | | | |  |
|  | *Diplazium amamianum* Tagawa | 1 | Japan | Kagoshima Pref. | | TNS | 764119 | AB574961 | AB575599 | | | |  |
|  | *Diplazium chinense* (Baker) C.Chr. | 2 | Japan | Mie Pref. | | TNS | 764368 | AB574962 | AB575600 | | | |  |
|  | *Diplazium crassiusculum* Tardieu ex Ching | 2 | Japan | Okinawa Pref. | | TNS | 764013 | AB574963 | AB575601 | | | |  |
|  | *Diplazium deciduum* N.Ohta et M.Takamiya | 2 | Japan | Kagoshima Pref. | | TNS | 762601 | AB574964 | AB575602 | | | |  |
|  | *Diplazium dilatatum* Blume | 1 | Japan | Kagoshima Pref. | | TNS | 763364 | AB574965 | AB575603 | | | |  |
|  | *Diplazium doederleinii* (Luerss.) Makino | 3 | Japan | Kagoshima Pref. | | TNS | 764130 | AB574966 | AB575604 | | | |  |
|  | *Diplazium donianum* (Mett.) Tardieu var. *donianum* | 3 | Japan | Okinawa Pref. | | TNS | 759293 | AB574967 | AB575605 | | | |  |
|  | *Diplazium donianum* (Mett.) Tardieu var. *aphanoneuron* (Ohwi) Tagawa | 2 | Japan | Kagoshima Pref. | | TNS | 769197 | AB574968 | AB575606 | | | |  |
|  | *Diplazium esculentum* (Retz.) Sw. | 1 | Japan | Kagoshima Pref. | | TNS | 766501 | AB574969 | AB575607 | | | |  |
|  | *Diplazium fauriei* H.Christ | 2 | Japan | Wakayama Pref. | | TNS | 766475 | AB574970 | AB575608 | | | |  |
|  | *Diplazium griffithii* T.Moore | 2 | Japan | Kagoshima Pref. | | TNS | 764240 | AB574971 | AB575609 | | | |  |
|  | *Diplazium hachijoense* Nakai | 3 | Japan | Wakayama Pref. | | TNS | 766404 | AB574972 | AB575610 | | | |  |
|  | *Diplazium hayatamae* N.Ohta et M.Takamiya | 2 | Japan | Kagoshima Pref. | | TNS | 763239 | AB574973 | AB575611 | | | |  |
|  | *Diplazium incomptum* Tagawa | 2 | Japan | Okinawa Pref. | | TNS | 763907 | AB574974 | AB575612 | | | |  |
|  | *Diplazium kawakamii* Hayata | 3 | Japan | Kagoshima Pref. | | TNS | 763151 | AB574975 | AB575613 | | | |  |
|  | *Diplazium lobatum* (Tagawa) Tagawa | 3 | Japan | Okinawa Pref. | | TNS | 759358 | AB574976 | AB575614 | | | |  |
|  | *Diplazium longicarpum* Kodama | 2 | Japan | Tokyo Pref. | | TNS | 774847 | AB574977 | AB575615 | | | |  |
|  | *Diplazium mettenianum* (Miq.) C.Chr. | 2 | Japan | Shizuoka Pref. | | TNS | 763879 | AB574978 | AB575616 | | | |  |
|  | *Diplazium nipponicum* Tagawa | 2 | Japan | Kagoshima Pref. | | TNS | 763227 | AB574979 | AB575617 | | | |  |
|  | *Diplazium okudairae* Makino | 1 | Japan | Kagoshima Pref. | | TNS | 762604 | AB574980 | AB575618 | | | |  |
|  | *Diplazium pin-faense* Ching | 1 | Japan | Kumamoto Pref. | | TNS | 764381 | AB574981 | AB575619 | | | |  |
|  | *Diplazium pullingeri* (Baker) J.Sm.  (= *Monomelangium pullingeri* (Baker) Tagawa) | 2 | Japan | Kagoshima Pref. | | TNS | 764123 | AB574982 | AB575620 | | | |  |
|  | *Diplazium sibiricum* (Turcz. ex Kunze) Sa.Kurata var. *glabrum* (Tagawa) Sa.Kurata | 2 | Japan | Nagano Pref. | | TNS | 766633 | AB574983 | AB575621 | | | |  |
|  | *Diplazium squamigerum* (Mett.) *Matsum*. | 2 | Japan | Shizuoka Pref. | | TNS | 764354 | AB574984 | N/A | | | |  |
|  | *Diplazium subtripinnatum* Nakai | 2 | Japan | Tokyo Pref. | | TNS | 743675 | AB574985 | N/A | | | |  |
|  | *Diplazium taiwanense* Tagawa | 3 | Japan | Kagoshima Pref. | | TNS | 763347 | AB574986 | AB575622 | | | |  |
|  | *Diplazium virescens* Kunze var. *virescens* | 3 | Japan | Kagoshima Pref. | | TNS | 763394 | AB574987 | AB575623 | | | |  |
|  | *Diplazium virescens* Kunze var. *conterminum* (H.Christ) Sa.Kurata | 3 | Japan | Miyazaki Pref. | | TNS | 762764 | AB574988 | AB575624 | | | |  |
|  | *Diplazium virescens* Kunze var. *okinawaense* (Tagawa) Sa.Kurata | 3 | Japan | Wakayama Pref. | | TNS | 766416 | AB574989 | AB575625 | | | |  |
|  | *Diplazium wichurae* (Mett.) Diels var. *wichurae* | 1 | Japan | Miyazaki Pref. | | TNS | 762722 | AB574990 | AB575626 | | | |  |
|  | *Diplazium wichurae* (Mett.) Diels var. *amabile* Tagawa | 1 | Japan | Kochi Pref. | | TNS | 771535 | AB574991 | AB575627 | | | |  |
|  | *Gymnocarpium dryopteris* (L.) Newman var. *dryopteris* | 0 | Japan | Hokkaido Pref. | | TNS | 765695 | AB574992 | AB575628 | | | |  |
|  | *Gymnocarpium dryopteris* (L.) Newman var. *aokigaharaense* Nakaike | 0 | Japan | Yamanashi Pref. | | TNS | 776978 | AB574993 | AB575629 | | | |  |
|  | *Gymnocarpium jessoense* (Koidz.) Koidz. | 1 | Japan | Iwate Pref. | | TNS | 765909 | AB574994 | AB575630 | | | |  |
|  | *Gymnocarpium oyamense* (Baker) Ching | 2 | Japan | Tokyo Pref. | | TNS | 769196 | AB574995 | AB575631 | | | |  |
|  | *Rhachidosorus mesosorus* (Makino) Ching  (= *Diplazium mesosorum* (Makino) Koidz.) | 1 | Japan | Saitama Pref. | | TNS | 764363 | AB574996 | AB575632 | | | |  |
|  | *Woodsia glabella* R.Br. ex Richards. | 1 | Japan | Saitama Pref. | | TNS | 776411 | AB574997 | AB575633 | | | |  |
|  | *Woodsia ilvensis* (L.) R.Br. | 0 | Japan | Hokkaido Pref. | | TNS | 765682 | AB574998 | AB575634 | | | |  |
|  | *Woodsia intermedia* Tagawa | 2 | Japan | Hiroshima Pref. | | TNS | 743747 | AB574999 | N/A | | | |  |
|  | *Woodsia macrochlaena* Mett. ex Kuhn | 1 | Japan | Kochi Pref. | | TNS | 764372 | AB575000 | AB575635 | | | |  |
|  | *Woodsia manchuriensis* Hook. | 1 | Japan | Saitama Pref. | | TNS | 764371 | AB575001 | AB575636 | | | |  |
|  | *Woodsia polystichoides* D.C.Eaton | 1 | Japan | Yamagata Pref. | | TNS | 765253 | AB575002 | AB575637 | | | |  |
|  | *Woodsia subcordata* Turcz. | 2 | Japan | Hokkaido Pref. | | TNS | 776980 | AB575003 | N/A | | | |  |
| Thelypteridaceae | |  |  |  | |  |  |  |  | | | |  |
|  | *Stegnogramma griffithii* (Hook.f. et Thomson) K.Iwats. var. *wilfordii* (Hook.) K.Iwats.  (= *Dictyocline griffithii* (Hook.f. et Thomson) T.Moore var. *wilfordii* (Hook.) T.Moore) | 2 | Japan | Mie Pref. | | TNS | 766423 | AB575004 | AB575638 | | | |  |
|  | *Stegnogramma gymnocarpa* (Copel.) K.Iwats. subsp. *amabilis* (Tagawa) K.Iwats. | 1 | Japan | Kochi Pref. | | TNS | 764388 | AB575005 | AB575639 | | | |  |
|  | *Stegnogramma pozoi* (Lag.) K.Iwats. subsp. *mollissima* (Fisch. ex Kunze) K.Iwats. | 1 | Japan | Fukuoka Pref. | | TNS | 763640 | AB575006 | AB575640 | | | |  |
|  | *Thelypteris acuminata* (Houtt.) C.V.Morton  (= *Christella acuminata* (Houtt.) H.Lév.) | 1 | Japan | Shizuoka Pref. | | TNS | 765135 | AB575007 | AB575641 | | | |  |
|  | *Thelypteris angulariloba* Ching  (= *Parathelypteris angulariloba* (Ching) Ching) | 2 | Japan | Okinawa Pref. | | TNS | 764017 | AB575008 | AB575642 | | | |  |
|  | *Thelypteris angustifrons* (Miq.) Ching  (= *Parathelypteris angustifrons* (Miq.) Ching) | 2 | Japan | Miyazaki Pref. | | TNS | 762751 | AB575009 | AB575643 | | | |  |
|  | *Thelypteris aurita* (Hook.) Ching  (= *Pseudophegopteris aurita* (Hook.) Ching) | 1 | Japan | Fukuoka Pref. | | TNS | 774838 | AB575010 | AB575644 | | | |  |
|  | *Thelypteris beddomei* (Baker) Ching  (= *Lastrea beddomei* (Baker) Bedd.) | 1 | Japan | Shizuoka Pref. | | TNS | 766446 | AB575011 | AB575645 | | | |  |
|  | *Thelypteris boninensis* (Kodama ex Koidz.) K.Iwats.  (= *Christella boninensis* (Kodama ex Koidz.) Holttum) |  | Japan | Tokyo Pref. | | TNS | 765140 | AB575012 | AB575646 | | | |  |
|  | *Thelypteris bukoensis* (Tagawa) Ching  (= *Pseudophegopteris bukoensis* (Tagawa) Holttum) | 1 | Japan | Gifu Pref. | | TNS | 743783 | AB575013 | N/A | | | |  |
|  | *Thelypteris castanea* (Tagawa) Ching  (= *Parathelypteris castanea* (Tagawa) Ching) | 0 | Japan | Okinawa Pref. | | TNS | 759279 | AB575014 | AB575647 | | | |  |
|  | *Thelypteris cystopteroides* (D.C.Eaton) Ching  (= *Parathelypteris cystopteroides* (D.C.Eaton) Ching) | 2 | Japan | Miyazaki Pref. | | TNS | 762749 | AB575015 | AB575648 | | | |  |
|  | *Thelypteris decursivepinnata* (H.C.Hall) Ching  (= *Phegopteris decursivepinnata* (H.C.Hall) Fée) | 2 | Japan | Ibaraki Pref. | | TNS | 764336 | AB575016 | AB575649 | | | |  |
|  | *Thelypteris dentata* (Forssk.) E.P.St.John  (= *Christella dentata* (Forssk.) Brownsey et Jermy) | 0 | Taiwan |  | | TNS | 763878 | AB575017 | AB575650 | | | |  |
|  | *Thelypteris erubescens* (Wall. ex Hook.) Ching  (= *Glaphyropteridopsis erubescens* (Wall. ex Hook.) Ching) | 0 | Japan | Kumamoto Pref. | | TNS | 1107832 | AB575018 | N/A | | | |  |
|  | *Thelypteris esquirolii* (H.Christ) Ching var. *glabrata* (H.Christ) K.Iwats. | 1 | Japan | Miyazaki Pref. | | TNS | 762732 | AB575019 | AB575651 | | | |  |
|  | *Thelypteris flexilis* (H.Christ) Ching  (= *Cyclogramma flexile* (H.Christ) Tagawa) | 2 | Japan | Kochi Pref. | | TNS | 763863 | AB575020 | AB575652 | | | |  |
|  | *Thelypteris glanduligera* (Kunze) Ching  (= *Parathelypteris glanduligera* (Kunze) Ching) | 2 | Japan | Kanagawa Pref. | | TNS | 763997 | AB575021 | AB575653 | | | |  |
|  | *Thelypteris gracilescens* (Blume) Ching  (= *Metathelypteris gracilescens* (Blume) Ching) | 0 | Japan | Kagoshima Pref. | | TNS | 763184 | AB575022 | AB575654 | | | |  |
|  | *Thelypteris hattorii* (H.Itô) Tagawa var. *hattorii*  (= *Metathelypteris hattorii* (H.Itô) Ching) | 0 | Japan | Kochi Pref. | | TNS | 768182 | AB575023 | AB575655 | | | |  |
|  | *Thelypteris hattorii* (H.Itô) Tagawa var. *nemoralis* (Ching) Sa.Kurata  (= *Metathelypteris hattorii* (H.Itô) Ching var. *nemoralis* (Ching) Nakaike) | 0 | Japan | Aichi Pref. | | TNS | 743782 | AB575024 | N/A | | | |  |
|  | *Thelypteris interrupta* (Willd.) K.Iwats.  (= *Cyclosorus interruptus* (Willd.) H. Itô) | 1 | Japan | Kagoshima Pref. | | TNS | 764370 | AB575025 | AB575656 | | | |  |
|  | *Thelypteris jaculosa* (C.Chr.) Panigr.  (= *Christella jaculosa* (C.Chr.) Holttum) |  | Japan | Kagoshima Pref. | | TNS | 764228 | AB575026 | AB575657 | | | |  |
|  | *Thelypteris japonica* (Baker) Ching  (= *Parathelypteris japonica* (Baker) Ching) | 2 | Japan | Tokyo Pref. | | TNS | 776368 | AB575027 | AB575658 | | | |  |
|  | *Thelypteris laxa* (Franch. et Sav.) Ching  (= *Metathelypteris laxa* (Franch. et Sav.) Ching) | 2 | Japan | Kyoto Pref. | | TNS | 763918 | AB575028 | AB575659 | | | |  |
|  | *Thelypteris liukiuensis* (H.Christ ex Matsum.) K.Iwats.  (= *Pronephrium liukiuense* (H.Christ ex Matsum.) Nakaike) | 2 | Japan | Okinawa Pref. | | TNS | 759270 | AB575029 | AB575660 | | | |  |
|  | *Thelypteris miyagii* (H.Itô) Nakato, Sahashi et M.Kato | 1 | Japan | Okinawa Pref. | | TNS | 1107836 | AB575030 | AB575661 | | | |  |
|  | *Thelypteris musashiensis* (Hiyama) Nakato, Sahashi et M.Kato | 1 | Japan | Saitama Pref. | | TNS | 776398 | AB575031 | AB575662 | | | |  |
|  | *Thelypteris nipponica* (Franch. et Sav.) Ching var. *nipponica*  (= *Parathelypteris nipponica* (Franch. et Sav.) Ching) | 2 | Japan | Nagano Pref. | | TNS | 766632 | AB575032 | AB575663 | | | |  |
|  | *Thelypteris nipponica* (Franch. et Sav.) Ching var. *borealis* (H.Hara) H.Hara | 0 | Japan | Yamanashi Pref. | | TNS | 776977 | AB575033 | AB575664 | | | |  |
|  | *Thelypteris ogasawarensis* (Nakai) H.Itô ex Honda  (= *Macrothelypteris ogasawarensis* (Nakai) Holttum) | 1 | Japan | Tokyo Pref. | | TNS | 764376 | AB575034 | AB575665 | | | |  |
|  | *Thelypteris omeiensis* (Baker) Ching  (= *Cyclogramma omeiense* (Baker) Tagawa) | 2 | Japan | Mie Pref. | | TNS | 763945 | AB575035 | AB575666 | | | |  |
|  | *Thelypteris palustris* (Salisb.) Schott | 1 | Japan | Ibaraki Pref. | | TNS | 769190 | AB575036 | AB575667 | | | |  |
|  | *Thelypteris parasitica* (L.) Tardieu  (= *Christella parasitica* (L.) H.Lév.) | 2 | Japan | Kagoshima Pref. | | TNS | 763344 | AB575037 | AB575668 | | | |  |
|  | *Thelypteris phegopteris* (L.) Sloss. ex Rydb.  (= *Phegopteris connectilis* (Michx.) Watt) | 3 | Japan | Akita Pref. | | TNS | 765160 | AB575038 | AB575669 | | | |  |
|  | *Thelypteris quelpaertensis* (H.Christ) Ching  (= *Lastrea quelpaertensis* (H.Christ) Copel.) | 1 | Japan | Akita Pref. | | TNS | 765773 | AB575039 | AB575670 | | | |  |
|  | *Thelypteris simplex* (Hook.) K.Iwats.  (= *Pronephrium simplex* (Hook.) Holttum) | 2 | Japan | Okinawa Pref. | | TNS | 759362 | AB575040 | AB575671 | | | |  |
|  | *Thelypteris subaurita* (Tagawa) Ching  (= *Pseudophegopteris subaurita* (Tagawa) Ching) | 1 | Japan | Kagoshima Pref. | | TNS | 764195 | AB575041 | AB575672 | | | |  |
|  | *Thelypteris taiwanensis* (C.Chr.) K.Iwats.  (= *Sphaerostephanos taiwanensis* (C.Chr.) Holttum ex C.M.Kuo) | 2 | Japan | Okinawa Pref. | | TNS | 759356 | AB575042 | AB575673 | | | |  |
|  | *Thelypteris torresiana* (Gaudich.) Alston var. *torresiana*  (= *Macrothelypteris torresiana* (Gaudich.) Ching) | 2 | Japan | Okinawa Pref. | | TNS | 759258 | AB575043 | AB575674 | | | |  |
|  | *Thelypteris torresiana* (Gaudich.) Alston var. *calvata* (Baker) K.Iwats.  (= *Macrothelypteris torresiana* (Gaudich.) Ching var. *calvata* (Baker) Holttum) | 2 | Japan | Kagoshima Pref. | | TNS | 763418 | AB575044 | AB575675 | | | |  |
|  | *Thelypteris triphylla* (Sw.) K.Iwats. var. *triphylla*  (= *Pronephrium triphyllum* (Sw.) Holttum) | 2 | Japan | Kagoshima Pref. | | TNS | 764187 | AB575045 | AB575676 | | | |  |
|  | *Thelypteris triphylla* (Sw.) K.Iwats. var. *parishii* (Bedd.) K.Iwats.  (= *Pronephrium triphyllum* (Sw.) Holttum var. *parishii* (Bedd.) Nakaike) | 2 | Japan | Okinawa Pref. | | TNS | 770336 | AB575046 | AB575677 | | | |  |
|  | *Thelypteris truncata* (Poir.) K.Iwats.  (= *Pneumatopteris truncata* (Poir.) Holttum) | 2 | Japan | Okinawa Pref. | | TNS | 770192 | AB575047 | N/A | | | |  |
|  | *Thelypteris uraiensis* (Rosenst.) Ching  (= *Metathelypteris uraiensis* (Rosenst.) Ching) | 2 | Japan | Kagoshima Pref. | | TNS | 763936 | AB575048 | AB575678 | | | |  |
|  | *Thelypteris viridifrons* Tagawa  (= *Macrothelypteris viridifrons* (Tagawa) Ching) | 2 | Japan | Ibaraki Pref. | | TNS | 766631 | AB575049 | AB575679 | | | |  |
| Blechnaceae | |  |  |  | |  |  |  |  | | | |  |
|  | *Blechnum amabile* Makino  (= *Struthiopteris amabilis* (Makino) Ching) | 1 | Japan | Kagoshima Pref. | | TNS | 763339 | AB575050 | AB575680 | | | |  |
|  | *Blechnum castaneum* Makino  (= *Struthiopteris castanea* (Makino) Nakai) | 2 | Japan | Akita Pref. | | TNS | 765789 | AB575051 | AB575681 | | | |  |
|  | *Blechnum hancockii* Hance  (= *Struthiopteris hancockii* (Hance) Tagawa) | 0 | Taiwan |  | | TNS | 776516 | AB575052 | AB575682 | | | |  |
|  | *Blechnum niponicum* (Kunze) Makino var. *Nipponicum*  (= *Struthiopteris niponica* (Kunze) Nakai) | 1 | Japan | Kagoshima Pref. | | TNS | 773471 | AB575053 | AB575683 | | | |  |
|  | *Blechnum niponicum* (Kunze) Makino var. *minimum* (Tagawa) Tagawa ex K.Iwats.  (= *Struthiopteris niponica* (Kunze) Nakai var. *minima* (Tagawa) Masam.) | 1 | Japan | Kagoshima Pref. | | TNS | 763250 | AB575054 | AB575684 | | | |  |
|  | *Blechnum orientale* L. | 1 | Japan | Okinawa Pref. | | TNS | 759298 | AB575055 | AB575685 | | | |  |
|  | *Woodwardia harlandii* Hook. | 0 | Japan | Okinawa Pref. | | TNS | 777850 | AB575056 | N/A | | | |  |
|  | *Woodwardia japonica* (L.f.) J.Sm. | 1 | Japan | Kagoshima Pref. | | TNS | 762560 | AB575057 | N/A | | | |  |
|  | *Woodwardia kempii* Copel. | 2 | Japan | Kagoshima Pref. | | TNS | 773461 | AB575058 | AB575686 | | | |  |
|  | *Woodwardia orientalis* Sw. var. orientalis | 2 | Japan | Miyazaki Pref. | | TNS | 762697 | AB575059 | AB575687 | | | |  |
|  | Woodwardia orientalis Sw. var. *formosana* Rosenst. | 1 | Japan | Okinawa Pref. | | TNS | 759281 | AB575060 | AB575688 | | | |  |
|  | *Woodwardia unigemmata* (Makino) Nakai | 1 | Japan | Shizuoka Pref. | | TNS | 765120 | AB575061 | AB575689 | | | |  |
| Onocleaceae | |  |  |  | |  |  |  |  | | | |  |
|  | *Matteuccia struthiopteris* (L.) Tod. | 1 | Japan | Ibaraki Pref. | | TNS | 768167 | AB575062 | AB575690 | | | |  |
|  | *Onoclea orientalis* (Hook.) Hook.  (= *Matteuccia orientalis* (Hook.) Trevis.) | 1 | Japan | Ibaraki Pref. | | TNS | 9508217 | AB575063 | AB575691 | | | |  |
|  | *Onoclea sensibilis* L. var. *interrupta* Maxim. | 1 | Japan | Akita Pref. | | TNS | 765732 | AB575064 | AB575692 | | | |  |
| Dryopteridaceae | |  |  |  | |  |  |  |  | | | |  |
|  | *Acrophorus nodosus* C.Presl | 1 | Japan | Kagoshima Pref. | | TNS | 763152 | AB575065 | AB575693 | | | |  |
|  | *Arachniodes amabilis* (Blume) Tindale var. *amabilis* | 1 | Japan | Miyazaki Pref. | | TNS | 762745 | AB575066 | AB575694 | | | |  |
|  | *Arachniodes amabilis* (Blume) Tindale var. *okinawensis* (Nakaike) Seriz. | 1 | Japan | Okinawa Pref. | | TNS | 764011 | AB575067 | AB575695 | | | |  |
|  | *Arachniodes amabilis* (Blume) Tindale var. *yakusimensis* (H.Itô) Ohwi | 2 | Japan | Kagoshima Pref. | | TNS | 763053 | AB575068 | AB575696 | | | |  |
|  | *Arachniodes aristata* (G.Forst.) Tindale | 1 | Japan | Kagoshima Pref. | | TNS | 763061 | AB575069 | AB575697 | | | |  |
|  | *Arachniodes borealis* Seriz. | 1 | Japan | Yamagata Pref. | | TNS | 765231 | AB575070 | AB575698 | | | |  |
|  | *Arachniodes cantilenae* Sa.Kurata | 1 | Japan | Kumamoto Pref. | | TNS | 762687 | AB575071 | AB575699 | | | |  |
|  | *Arachniodes cavaleriei* (H.Christ) Ohwi | 1 | Japan | Kagoshima Pref. | | TNS | 736794 | AB575072 | AB575700 | | | |  |
|  | *Arachniodes chinensis* (Rosenst.) Ching | 1 | Japan | Kagoshima Pref. | | TNS | 771429 | AB575073 | AB575701 | | | |  |
|  | *Arachniodes davalliiformis* (H.Christ) Nakaike | 2 | Japan | Tokyo Pref. | | TNS | 763897 | AB575074 | AB575702 | | | |  |
|  | *Arachniodes dimorphophylla* (Hayata) Ching | 1 | Japan | Okinawa Pref. | | TNS | 763895 | AB575075 | AB575703 | | | |  |
|  | *Arachniodes hekiana* Sa.Kurata | 1 | Japan | Kagoshima Pref. | | TNS | 763884 | AB575076 | AB575704 | | | |  |
|  | *Arachniodes hiugana* Sa.Kurata | 1 | Japan | Miyazaki Pref. | | TNS | 1107831 | AB575077 | N/A | | | |  |
|  | *Arachniodes miqueliana* Ohwi  (= *Leptorumohra miqueliana* (Maxim.) H.Itô) | 2 | Japan | Kyoto Pref. | | TNS | 763865 | AB575078 | AB575705 | | | |  |
|  | *Arachniodes mutica* (Franch. et Sav.) Ohwi | 2 | Japan | Yamanashi Pref. | | TNS | 763931 | AB575079 | AB575706 | | | |  |
|  | *Arachniodes nipponica* (Rosenst.) Ohwi | 1 | Japan | Shizuoka Pref. | | TNS | 9508318 | AB575080 | AB575707 | | | |  |
|  | *Arachniodes oohorae* H.Itô | 1 | Japan | Mie Pref. | | TNS | 766442 | AB575081 | AB575708 | | | |  |
|  | *Arachniodes simplicior* (Makino) Ohwi var. *simplicior* | 2 | Locality uncertain |  | | TNS | 9508131 | AB575082 | AB575709 | | | |  |
|  | *Arachniodes simplicior* (Makino) Ohwi var. *major* (Tagawa) Ohwi | 2 | Japan | Shizuoka Pref. | | TNS | 763906 | AB575083 | AB575710 | |  | |  |
|  | *Arachniodes sinomiqueliana* (Ching) Ohwi  (= *Leptorumohra sinomiqueliana* (Ching) Tagawa) | 0 | Japan | | Kumamoto Pref. | TNS | 762679 | AB575084 | AB575711 | |  | |  |
|  | *Arachniodes sporadosora* (Kunze) Nakaike | 1 | Japan | | Kagoshima Pref. | TNS | 763474 | AB575085 | AB575712 | |  | |  |
|  | *Arachniodes standishii* (T.Moore) Ohwi | 1 | Japan | | Kagoshima Pref. | TNS | 762593 | AB575086 | AB575713 | |  | |  |
|  | *Arachniodes yasu-inouei* Sa.Kurata | 2 | Japan | | Mie Pref. | TNS | 766431 | AB575087 | N/A | |  | |  |
|  | *Arachniodes yoshinagae* (Makino) Ching | 2 | Japan | | Miyazaki Pref. | TNS | 762779 | AB575088 | AB575714 | |  | |  |
|  | *Bolbitis appendiculata* (Willd.) K.Iwats. | 0 | Japan | | Okinawa Pref. | TNS | 759269 | AB575089 | AB575715 | |  | |  |
|  | *Bolbitis heteroclita* (C.Presl) Ching | 1 | Japan | | Okinawa Pref. | TNS | 764366 | AB575090 | AB575716 | |  | |  |
|  | *Bolbitis quoyana* (Gaudich.) Ching | 1 | Japan | | Tokyo Pref. | TNS | 764358 | AB575091 | AB575717 | |  | |  |
|  | *Bolbitis subcordata* (Copel.) Ching | 1 | Japan | | Okinawa Pref. | TNS | 764012 | AB575092 | AB575718 | |  | |  |
|  | *Ctenitis eatonii* (Baker) Ching | 0 | Japan | | Kagoshima Pref. | TNS | 764233 | AB575093 | AB575719 | |  | |  |
|  | *Ctenitis iriomotensis* (H.Itô) Nakaike | 1 | Japan | | Okinawa Pref. | TNS | 763919 | AB575094 | AB575720 | |  | |  |
|  | *Ctenitis lepigera* (Baker) Tagawa | 1 | Japan | | Tokyo Pref. | TNS | 763898 | AB575095 | AB575721 | |  | |  |
|  | *Ctenitis maximowicziana* (Miq.) Ching  (= *Dryopsis maximowicziana* (Miq.) Holttum et Edwards) | 1 | Japan | | Shizuoka Pref. | TNS | 9508132 | AB575096 | N/A | |  | |  |
|  | *Ctenitis microlepigera* (Nakai) Ching | 0 | Japan | | Tokyo Pref. | TNS | 743678 | AB575097 | N/A | |  | |  |
|  | *Ctenitis sinii* (Ching) Ohwi | 1 | Japan | | Kumamoto Pref. | TNS | 762651 | AB575098 | AB575722 | |  | |  |
|  | *Ctenitis subglandulosa* (Hance) Ching | 1 | Japan | | Kagoshima Pref. | TNS | 763108 | AB575099 | AB575723 | |  | |  |
|  | *Cyrtomium caryotideum* (Wall. ex Hook. et Grev.) C.Presl | 3 | Nepal | |  | TNS | 771178 | AB575100 | AB575724 | |  | |  |
|  | *Cyrtomium devexiscapulae* (Koidz.) Ching | 2 | Japan | | Shizuoka Pref. | TNS | 771162 | AB575101 | AB575725 | |  | |  |
|  | *Cyrtomium falcatum* (L.f.) C.Presl subsp. *falcatum* | 3 | Japan | | Tokyo Pref. | TNS | 771172 | AB575102 | AB575726 | |  | |  |
|  | *Cyrtomium falcatum* (L.f.) C.Presl subsp. *australe* S.Matsumoto, nom. nud. | 1 | Japan | | Tokyo Pref. | TNS | 771177 | AB575103 | AB575727 | |  | |  |
|  | *Cyrtomium falcatum* (L.f.) C.Presl subsp. *littorale* S.Matsumoto, nom. nud. | 1 | Japan | | Shizuoka Pref. | TNS | 771160 | AB575104 | AB575728 | |  | |  |
|  | *Cyrtomium fortunei* J.Sm. var. *atropunctatum* (Sa.Kurata) K.Iwats. | 3 | Japan | | Shizuoka Pref. | TNS | 771164 | AB575105 | AB575729 | |  | |  |
|  | *Cyrtomium fortunei* J.Sm. var. *clivicola* (Makino) Tagawa | 3 | Japan | | Shizuoka Pref. | TNS | 771163 | AB575106 | AB575730 | |  | |  |
|  | *Cyrtomium fortunei* J.Sm. var. *intermedium* Tagawa | 3 | Japan | | Kanagawa Pref. | TNS | 771167 | AB575107 | AB575731 | |  | |  |
|  | *Cyrtomium laetevirens* (Hiyama) Nakaike | 3 | Japan | | Saitama Pref. | TNS | 771199 | AB575108 | AB575732 | |  | |  |
|  | *Cyrtomium macrophyllum* (Makino) Tagawa var. *macrophyllum* | 3 | Japan | | Kochi Pref. | TNS | 771173 | AB575109 | AB575733 | |  | |  |
|  | *Cyrtomium macrophyllum* (Makino) Tagawa var. *microindusium* (Sa.Kurata) K.Iwats. | 3 | Japan | | Kumamoto Pref. | TNS | 771166 | AB575110 | AB575734 | |  | |  |
|  | *Cyrtomium macrophyllum* (Makino) Tagawa var. *tukusicola* (Tagawa) Tagawa | 3 | Japan | | Fukuoka Pref. | TNS | 771200 | AB575111 | AB575735 | |  | |  |
|  | *Dryopteris amurensis* (Milde) H.Christ | 1 | Japan | | Hokkaido Pref. | TNS | 768171 | AB575112 | AB575736 | |  | |  |
|  | *Dryopteris anadroma* Mitsuta, nom. nud. | 0 | Japan | | Kagoshima Pref. | TNS | 763331 | AB575113 | AB575737 | |  | |  |
|  | *Dryopteris anthracinisquama* Miyam. | 3 | Japan | | Kumamoto Pref. | TNS | 9508645 | AB575114 | AB575738 | |  | |  |
|  | *Dryopteris atrata* (Wall. ex Kunze) Ching | 3 | Japan | | Kagoshima Pref. | TNS | 762592 | AB575115 | AB575739 | |  | |  |
|  | *Dryopteris bissetiana* (Baker) C.Chr. | 3 | Japan | | Kagoshima Pref. | TNS | 763335 | AB575116 | AB575740 | |  | |  |
|  | *Dryopteris caudipinna* Nakai | 1 | Japan | | Ibaraki Pref. | TNS | 764349 | AB575117 | AB575741 | |  | |  |
|  | *Dryopteris championii* (Benth.) C.Chr. ex Ching | 3 | Japan | | Gifu Pref. | TNS | 764357 | AB575118 | AB575742 | |  | |  |
|  | *Dryopteris chinensis* (Baker) Koidz. | 3 | Japan | | Tokyo Pref. | TNS | 763933 | AB575119 | AB575743 | |  | |  |
|  | *Dryopteris commixta* Tagawa | 2 | Japan | | Kumamoto Pref. | TNS | 763896 | AB575120 | AB575744 | |  | |  |
|  | *Dryopteris coreanomontana* Nakai | 1 | Japan | | Nagano Pref. | TNS | 763944 | AB575121 | AB575745 | |  | |  |
|  | *Dryopteris crassirhizoma* Nakai | 1 | Japan | | Nagano Pref. | TNS | 764333 | AB575122 | AB575746 | |  | |  |
|  | *Dryopteris decipiens* (Hook.) Kuntze var. *decipiens* | 3 | Japan | | Mie Pref. | TNS | 764382 | AB575123 | AB575747 | |  | |  |
|  | *Dryopteris decipiens* (Hook.) Kuntze var. *diplazioides* (H.Christ) Ching | 0 | Japan | | Nara Pref. | TNS | 766493 | AB575124 | AB575748 | |  | |  |
|  | *Dryopteris dickinsii* (Franch. et Sav.) C.Chr. | 3 | Japan | | Nara Pref. | TNS | 766491 | AB575125 | N/A | |  | |  |
|  | *Dryopteris erythrosora* (D.C.Eaton) Kuntze | 3 | Japan | | Kagoshima Pref. | TNS | 762595 | AB575126 | AB575749 | |  | |  |
|  | *Dryopteris expansa* (C.Presl) Fraser-Jenk. et Jermy | 1 | Japan | | Akita Pref. | TNS | 765180 | AB575127 | AB575750 | |  | |  |
|  | *Dryopteris formosana* (H.Christ) C.Chr. | 3 | Japan | | Kagoshima Pref. | TNS | 763153 | AB575128 | AB575751 | |  | |  |
|  | *Dryopteris fragrans* (L.) Schott | 1 | Japan | | Hokkaido Pref. | TNS | 743728 | AB575129 | N/A | |  | |  |
|  | *Dryopteris fuscipes* C.Chr. | 3 | Japan | | Kagoshima Pref. | TNS | 762541 | AB575130 | AB575752 | |  | |  |
|  | *Dryopteris gymnophylla* (Baker) C.Chr. | 1 | Japan | | Tokyo Pref. | TNS | 768169 | AB575131 | AB575753 | |  | |  |
|  | *Dryopteris gymnosora* (Makino) C.Chr. | 3 | Japan | | Kagoshima Pref. | TNS | 763325 | AB575132 | AB575754 | |  | |  |
|  | *Dryopteris hadanoi* Sa.Kurata | 3 | Japan | | Kochi Pref. | TNS | 763876 | AB575133 | AB575755 | |  | |  |
|  | *Dryopteris handeliana* C.Chr. | 1 | Japan | | Oita Pref. | TNS | 776385 | AB575134 | AB575756 | |  | |  |
|  | *Dryopteris hangchowensis* Ching | 1 | Japan | | Miyazaki Pref. | TNS | 9508643 | AB575135 | AB575757 | |  | |  |
|  | *Dryopteris hasseltii* (Blume) C.Chr. | 1 | Japan | | Okinawa Pref. | TNS | 777854 | AB575136 | AB575758 | |  | |  |
|  | *Dryopteris hayatae* Tagawa | 1 | Japan | | Kagoshima Pref. | TNS | 763060 | AB575137 | AB575759 | |  | |  |
|  | *Dryopteris hendersonii* (Bedd.) C.Chr. | 1 | Japan | | Kagoshima Pref. | TNS | 763159 | AB575138 | AB575760 | |  | |  |
|  | *Dryopteris hondoensis* Koidz. | 3 | Japan | | Shizuoka Pref. | TNS | 764343 | AB575139 | AB575761 | |  | |  |
|  | *Dryopteris indusiata* (Makino) Makino et Yamam. | 3 | Japan | | Kagoshima Pref. | TNS | 762629 | AB575140 | AB575762 | |  | |  |
|  | *Dryopteris insularis* Kodama var. *insularis* | 3 | Japan | | Tokyo Pref. | TNS | 1107830 | AB575141 | AB575763 | |  | |  |
|  | *Dryopteris insularis* Kodama var. *chichisimensis* (Nakai ex H.Itô) H.Itô | 3 | Japan | | Tokyo Pref. | TNS | 763914 | AB575142 | AB575764 | |  | |  |
|  | *Dryopteris intermedia* (Muhl. ex Willd.) A.Gray [introduced] | 0 | Japan | | Ibaraki Pref. | TNS | 766638 | AB575143 | AB575765 | |  | |  |
|  | *Dryopteris kinkiensis* Koidz. ex Tagawa | 2 | Japan | | Wakayama Pref. | TNS | 766399 | AB575144 | AB575766 | |  | |  |
|  | *Dryopteris kinokuniensis* Sa.Kurata | 0 | Japan | | Mie Pref. | TNS | 766432 | AB575145 | AB575767 | |  | |  |
|  | *Dryopteris koidzumiana* Tagawa | 1 | Japan | | Kagoshima Pref. | TNS | 763047 | AB575146 | AB575768 | |  | |  |
|  | *Dryopteris labordei* (H.Christ) C.Chr. var. *purpurascens* (H.Itô) Seriz. | 3 | Japan | | Kagoshima Pref. | TNS | 763324 | AB575147 | AB575769 | |  | |  |
|  | *Dryopteris lacera* (Thunb.) Kuntze | 1 | Japan | | Tokyo Pref. | TNS | 774850 | AB575148 | AB575770 | |  | |  |
|  | *Dryopteris laeta* (Kom.) C.Chr. | 1 | Japan | | Iwate Pref. | TNS | 765899 | AB575149 | AB575771 | |  | |  |
|  | *Dryopteris lunanensis* (H.Christ) C.Chr. | 1 | Japan | | Nara Pref. | TNS | 763899 | AB575150 | AB575772 | |  | |  |
|  | *Dryopteris maximowiczii* (Baker) Kuntze | 1 | Japan | | Shizuoka Pref. | TNS | 763866 | AB575151 | AB575773 | |  | |  |
|  | *Dryopteris medioxima* Koidz. | 3 | Japan | | Saitama Pref. | TNS | 776976 | AB575152 | AB575774 | |  | |  |
|  | *Dryopteris melanocarpa* Hayata | 3 | Japan | | Wakayama Pref. | TNS | 766410 | AB575153 | N/A | |  | |  |
|  | *Dryopteris monticola* (Makino) C.Chr. | 1 | Japan | | Yamagata Pref. | TNS | 765238 | AB575154 | AB575775 | |  | |  |
|  | *Dryopteris namegatae* (Sa.Kurata) Sa.Kurata | 3 | Japan | | Mie Pref. | TNS | 766467 | AB575155 | AB575776 | |  | |  |
|  | *Dryopteris nipponensis* Koidz. | 3 | Japan | | Kagoshima Pref. | TNS | 763145 | AB575156 | AB575777 | |  | |  |
|  | *Dryopteris pacifica* (Nakai) Tagawa | 3 | Japan | | Kagoshima Pref. | TNS | 763312 | AB575157 | AB575778 | |  | |  |
|  | *Dryopteris polita* Rosenst. | 1 | Japan | | Kagoshima Pref. | TNS | 763901 | AB575158 | AB575779 | |  | |  |
|  | *Dryopteris polylepis* (Franch. et Sav.) C.Chr. | 1 | Japan | | Nagano Pref. | TNS | 764340 | AB575159 | AB575780 | |  | |  |
|  | *Dryopteris pycnopteroides* (H.Christ) C.Chr. | 3 | Japan | | Shizuoka Pref. | TNS | 764373 | AB575160 | AB575781 | |  | |  |
|  | *Dryopteris ryo-itoana* Sa.Kurata | 3 | Japan | | Nara Pref. | TNS | 766494 | AB575161 | AB575782 | |  | |  |
|  | *Dryopteris sabae* (Franch. et Sav.) C.Chr. | 1 | Japan | | Ibaraki Pref. | TNS | 764337 | AB575162 | AB575783 | |  | |  |
|  | *Dryopteris sacrosancta* Koidz. | 3 | Japan | | Mie Pref. | TNS | 764377 | AB575163 | AB575784 | |  | |  |
|  | *Dryopteris saxifraga* H.Itô | 1 | Japan | | Ibaraki Pref. | TNS | 766451 | AB575164 | AB575785 | |  | |  |
|  | *Dryopteris saxifragivaria* Nakai | 0 | Japan | | Ibaraki Pref. | TNS | 763900 | AB575165 | AB575786 | |  | |  |
|  | *Dryopteris shibipedis* Sa.Kurata | 3 | Japan | | Kagoshima Pref. | TNS | 766463 | AB575166 | AB575787 | |  | |  |
|  | *Dryopteris shikokiana* (Makino) C.Chr. | 1 | Japan | | Kagoshima Pref. | TNS | 762596 | AB575167 | AB575788 | |  | |  |
|  | *Dryopteris shiroumensis* Sa.Kurata et T.Nakam. | 0 | Japan | | Nagano Pref. | TNS | 766466 | AB575168 | AB575789 | |  | |  |
|  | *Dryopteris sieboldii* (van Houtte ex Mett.) Kuntze | 2 | Japan | | Miyazaki Pref. | TNS | 762696 | AB575169 | AB575790 | |  | |  |
|  | *Dryopteris simasakii* (H.Itô) Sa.Kurata var. *simasakii* | 3 | Japan | | Nara Pref. | TNS | 766492 | AB575170 | AB575791 | |  | |  |
|  | *Dryopteris simasakii* (H.Itô) Sa.Kurata var. *paleacea* (H.Itô) Sa.Kurata | 3 | Japan | | Gifu Pref. | TNS | 766450 | AB575171 | AB575792 | |  | |  |
|  | *Dryopteris sordidipes* Tagawa | 1 | Japan | | Kagoshima Pref. | TNS | 763050 | AB575172 | AB575793 | |  | |  |
|  | *Dryopteris sparsa* (Buch.-Ham. ex D.Don) Kuntze | 3 | Japan | | Kagoshima Pref. | TNS | 763059 | AB575173 | AB575794 | |  | |  |
|  | *Dryopteris tokyoensis* (Makino) C.Chr. | 1 | Japan | | Ibaraki Pref. | TNS | 766452 | AB575174 | AB575795 | |  | |  |
|  | *Dryopteris tsugiwoi* Sa.Kurata | 3 | Japan | | Wakayama Pref. | TNS | 777859 | AB575175 | AB575796 | |  | |  |
|  | *Dryopteris tsutsuiana* Sa.Kurata | 3 | Japan | | Kumamoto Pref. | TNS | 743780 | AB575176 | N/A | |  | |  |
|  | *Dryopteris uniformis* (Makino) Makino | 2 | Japan | | Chiba Pref. | TNS | 774834 | AB575177 | AB575797 | |  | |  |
|  | *Dryopteris varia* (L.) Kuntze | 3 | Japan | | Wakayama Pref. | TNS | 763911 | AB575178 | AB575798 | |  | |  |
|  | *Dryopteris ebinoensis* Sa.Kurata, nom. nud. | 3 | Japan | | Kumamoto Pref. | TNS | 776383 | AB575179 | AB575799 | |  | |  |
|  | *Dryopteris yakusilvicola* Sa.Kurata | 3 | Japan | | Kagoshima Pref. | TNS | 763063 | AB575180 | AB575800 | |  | |  |
|  | *Elaphoglossum tosaense* (Yatabe) Makino | 0 | Japan | | Kagoshima Pref. | TNS | 763098 | AB575181 | AB575801 | |  | |  |
|  | *Elaphoglossum yoshinagae* (Yatabe) Makino | 0 | Japan | | Kagoshima Pref. | TNS | 763107 | AB575182 | AB575802 | |  | |  |
|  | *Hypodematium crenatum* (Forssk.) Kuhn subsp. *fauriei* (Kodama) K.Iwats. | 2 | Japan | | Kochi Pref. | TNS | 768177 | AB575183 | AB575803 | |  | |  |
|  | *Hypodematium* *fordii* (Baker) Ching | 0 | Locality uncertain | |  | TNS | 763905 | AB575184 | AB575804 | | | |  |
|  | *Hypodematium glandulosopilosum* (Tagawa) Ohwi | 0 | Japan | | Kochi Pref. | TNS | 768179 | AB575185 | AB575805 | |  | |  |
|  | *Polystichum balansae* H.Christ  (= *Cyrtomium balansae* (H.Christ) C.Chr.) | 1 | Japan | | Miyazaki Pref. | TNS | 771175 | AB575186 | N/A | |  | |  |
|  | *Polystichum braunii* (Spenn.) Fée subsp. *braunii* | 2 | Japan | | Hokkaido Pref. | TNS | 765677 | AB575187 | AB575806 | |  | |  |
|  | *Polystichum braunii* (Spenn.) Fée subsp. *kamtschaticum* (C.Chr. et Hultén) A. et D.Löve | 0 | Japan | | Nagano Pref. | TNS | 763942 | AB575188 | AB575807 | |  | |  |
|  | *Polystichum craspedosorum* (Maxim.) Diels | 1 | Japan | | Tokyo Pref. | TNS | 764000 | AB575189 | AB575808 | |  | |  |
|  | *Polystichum deltodon* (Baker) Diels | 2 | Japan | | Kumamoto Pref. | TNS | 762669 | AB575190 | AB575809 | |  | |  |
|  | *Polystichum eximium* (Mett. ex Kuhn) C.Chr. | 1 | Japan | | Kagoshima Pref. | TNS | 764380 | AB575191 | AB575810 | |  | |  |
|  | *Polystichum fibrillosopaleaceum* (Kodama) Tagawa var. *fibrillosopaleaceum* | 1 | Japan | | Shizuoka Pref. | TNS | 769191 | AB575192 | AB575811 | |  | |  |
|  | *Polystichum fibrillosopaleaceum* (Kodama) Tagawa var. *marginale* Seriz. | 1 | Japan | | Shizuoka Pref. | TNS | 738144 | AB575193 | N/A | |  | |  |
|  | *Polystichum formosanum* Rosenst | 1 | Locality uncertain | |  | TNS | 763920 | AB575194 | | AB575812 | |  | |
|  | *Polystichum gracilipes* C.Chr. var. *gemmiferum* Tagawa | 0 | Japan | | Nagano Pref. | TNS | 776367 | AB575195 | AB575813 | |  | |  |
|  | *Polystichum grandifrons* C.Chr. | 3 | Japan | | Kagoshima Pref. | TNS | 771415 | AB575196 | AB575814 | |  | |  |
|  | *Polystichum hancockii* (Hance) Diels | 1 | Japan | | Okinawa Pref. | TNS | 763908 | AB575197 | AB575815 | |  | |  |
|  | *Polystichum hookerianum* (C.Presl) C.Chr.  (= *Cyrtomium* *hookerianum* (C.Presl) C.Chr.) | 1 | Japan | | Kagoshima Pref. | TNS | 771431 | AB575198 | AB575816 | |  | |  |
|  | *Polystichum igaense* Tagawa | 1 | Japan | | Tokyo Pref. | TNS | 763928 | AB575199 | AB575817 | |  | |  |
|  | *Polystichum inaense* (Tagawa) Tagawa | 1 | Japan | | Nagano Pref. | TNS | 776482 | AB575200 | AB575818 | |  | |  |
|  | *Polystichum lachenense* (Hook.) Bedd. | 0 | Japan | | Nagano Pref. | TNS | 766487 | AB575201 | AB575819 | |  | |  |
|  | *Polystichum lepidocaulon* (Hook.) J.Sm. | 1 | Japan | | Tokyo Pref. | TNS | 764341 | AB575202 | AB575820 | |  | |  |
|  | *Polystichum lonchitis* (L.) Roth ex Roem. | 0 | France | |  | TNS | 743763 | AB575203 | N/A | |  | |  |
|  | *Polystichum longifrons* Sa.Kurata | 2 | Japan | | Ibaraki Pref. | TNS | 774851 | AB575204 | AB575821 | |  | |  |
|  | *Polystichum makinoi* (Tagawa) Tagawa | 2 | Japan | | Kanagawa Pref. | TNS | 764342 | AB575205 | AB575822 | |  | |  |
|  | *Polystichum microchlamys* (H.Christ) Matsum. var. *microchlamys* | 1 | Japan | | Akita Pref. | TNS | 765776 | AB575206 | AB575823 | |  | |  |
|  | *Polystichum microchlamys* (H.Christ) Matsum. var. *azumiense* Seriz. | 2 | Japan | | Akita Pref. | TNS | 765798 | AB575207 | N/A | |  | |  |
|  | *Polystichum neolobatum* Nakai | 3 | Japan | | Nagano Pref. | TNS | 743679 | AB575208 | N/A | |  | |  |
|  | *Polystichum obae* Tagawa | 1 | Japan | | Kagoshima Pref. | TNS | 766461 | AB575209 | AB575824 | |  | |  |
|  | *Polystichum ohmurae* Sa.Kurata | 1 | Japan | | Nagano Pref. | TNS | 766490 | AB575210 | AB575825 | |  | |  |
|  | *Polystichum otomasui* Sa.Kurata | 1 | Japan | | Miyazaki Pref. | TNS | 762702 | AB575211 | AB575826 | |  | |  |
|  | *Polystichum ovatopaleaceum* (Kodama) Sa.Kurata | 2 | Japan | | Shizuoka Pref. | TNS | 9508214 | AB575212 | AB575827 | |  | |  |
|  | *Polystichum ovatopaleaceum* (Kodama) Sa.Kurata var. *coraiense* (H.Christ ex H.Lév.) Sa.Kurata | 2 | Japan | | Ibaraki Pref. | TNS | 764355 | AB575213 | AB575828 | |  | |  |
|  | *Polystichum piceopaleaceum* Tagawa | 0 | Nepal | |  | TNS | 763912 | AB575214 | AB575829 | |  | |  |
|  | *Polystichum polyblepharon* (Roem. ex Kunze) C.Presl var. *polyblepharon* | 2 | Japan | | Kagoshima Pref. | TNS | 762608 | AB575215 | AB575830 | |  | |  |
|  | *Polystichum polyblepharon* (Roem. ex Kunze) C.Presl var. *scabiosum* Sa.Kurata | 2 | Japan | | Chiba Pref. | TNS | 764356 | AB575216 | AB575831 | |  | |  |
|  | *Polystichum pseudomakinoi* Tagawa | 2 | Locality uncertain | | TNS | 763861 | AB575217 | AB575832 |  | | | |  |
|  | *Polystichum retrosopaleaceum* (Kodama) Tagawa | 1 | Japan | | Kyoto Pref. | TNS | 9508176 | AB575218 | AB575833 | |  | |  |
|  | *Polystichum rigens* Tagawa | 3 | Japan | | Tokyo Pref. | TNS | 764383 | AB575219 | AB575834 | |  | |  |
|  | *Polystichum shimurae* Sa.Kurata ex Seriz. | 2 | Japan | | Tokyo Pref. | TNS | 763885 | AB575220 | AB575835 | |  | |  |
|  | *Polystichum tagawanum* Sa.Kurata | 2 | Japan | | Shizuoka Pref. | TNS | 9508172 | AB575221 | AB575836 | |  | |  |
|  | *Polystichum tripteron* (Kunze) C.Presl | 1 | Japan | | Kagoshima Pref. | TNS | 763162 | AB575222 | AB575837 | |  | |  |
|  | *Polystichum tsus-simense* (Hook.) J.Sm. var. *tsus-simense* | 3 | Japan | | Kumamoto Pref. | TNS | 762664 | AB575223 | AB575838 | |  | |  |
|  | *Polystichum tsus-simense* (Hook.) J.Sm. var. *mayebarae* (Tagawa) Sa.Kurata | 3 | Japan | | Tokyo Pref. | TNS | 765125 | AB575224 | AB575839 | |  | |  |
|  | *Polystichum yaeyamense* (Makino) Makino | 1 | Japan | | Okinawa Pref. | TNS | 759286 | AB575225 | AB575840 | |  | |  |
| Lomariopsidaceae | |  |  | |  |  |  |  |  | |  | |  |
|  | *Lomariopsis spectabilis* (Kunze) Mett. | 0 | Japan | | Tokyo Pref. | TNS | 763923 | AB575226 | AB575841 | |  | |  |
|  | *Nephrolepis biserrata* (Sw.) Schott | 0 | Japan | | Okinawa Pref. | TNS | 759318 | AB575227 | N/A | |  | |  |
|  | *Nephrolepis cordifolia* (L.) C.Presl | 1 | Japan | | Kagoshima Pref. | TNS | 763485 | AB575228 | AB575842 | |  | |  |
|  | *Nephrolepis hirsutula* (G.Forst.) C.Presl | 0 | Japan | | Okinawa Pref. | TNS | 768166 | AB575229 | AB575843 | |  | |  |
| Tectariaceae | |  |  | |  |  |  |  |  | |  | |  |
|  | *Arthropteris palisotii* (Desv.) Alston | 0 | Japan | | Okinawa Pref. | TNS | 763921 | AB575230 | AB575844 | |  | |  |
|  | *Hemigramma decurrens* (Hook.) Copel. | 2 | Japan | | Okinawa Pref. | TNS | 759275 | AB575231 | AB575845 | |  | |  |
|  | *Tectaria decurrens* (C.Presl) Copel. | 0 | Japan | | Okinawa Pref. | TNS | 759312 | AB575232 | AB575846 | |  | |  |
|  | *Tectaria devexa* (Kunze) Copel. | 0 | Japan | | Okinawa Pref. | TNS | 764369 | AB575233 | AB575847 | |  | |  |
|  | *Tectaria fauriei* Tagawa | 0 | Japan | | Kagoshima Pref. | TNS | 764229 | AB575234 | AB575848 | |  | |  |
|  | *Tectaria phaeocaulis* (Rosenst.) C.Chr. | 0 | Taiwan | |  | TNS | 743677 | AB575235 | N/A | |  | |  |
|  | *Tectaria simonsii* (Bedd.) Ching | 0 | Japan | | Okinawa Pref. | TNS | 743680 | AB575236 | N/A | |  | |  |
|  | *Tectaria subtriphylla* (Hook. et Arn.) Copel. | 1 | Japan | | Okinawa Pref. | TNS | 759338 | AB575237 | AB575849 | |  | |  |
| Davalliaceae | |  |  | |  |  |  |  |  | |  | |  |
|  | *Davallia mariesii* T.Moore ex Baker | 1 | Japan | | Kagoshima Pref. | TNS | 763309 | AB575238 | AB575850 | |  | |  |
|  | *Humata repens* (L.f.) Diels  (= *Pachypleuria repens* (L.f.) M.Kato) | 0 | Japan | | Kagoshima Pref. | TNS | 763306 | AB575239 | AB575851 | |  | |  |
|  | *Humata trifoliata* Cav.  (= *Pachypleuria trifoliata* (Cav.) C.Presl) | 0 | Japan | | Okinawa Pref. | TNS | 759330 | AB575240 | AB575852 | |  | |  |
| Polypodiaceae | |  |  | |  |  |  |  |  | |  | |  |
|  | *Aglaomorpha coronans* (Wall. ex Mett.) Copel. (= *Pseudodrynaria coronans* (Wall. ex Mett.) Ching) | 0 | Japan | | Okinawa Pref. | TNS | 770271 | AB575241 | AB575853 | |  | |  |
|  | *Colysis elegans* Sa.Kurata | 0 | Japan | | Kagoshima Pref. | TNS | 774825 | AB575242 | AB575854 | |  | |  |
|  | *Colysis elliptica* (Thunb.) Ching | 1 | Japan | | Mie Pref. | TNS | 774822 | AB575243 | AB575855 | |  | |  |
|  | *Colysis hemionitidea* C.Presl | 0 | Japan | | Kagoshima Pref. | TNS | 763475 | AB575244 | AB575856 | |  | |  |
|  | *Colysis pothifolia* (Buch.-Ham. ex D.Don) C.Presl | 1 | Japan | | Tokyo Pref. | TNS | 774819 | AB575245 | AB575857 | |  | |  |
|  | *Colysis pteropus* (Blume) Bosman  (= *Microsorum pteropus* (Blume) Copel.) | 2 | Japan | | Okinawa Pref. | TNS | 759285 | AB575246 | AB575858 | |  | |  |
|  | *Colysis wrightii* (Hook.) Ching | 1 | Japan | | Kagoshima Pref. | TNS | 774817 | AB575247 | N/A | |  | |  |
|  | *Crypsinus engleri* (Luerss.) Copel. | 1 | Japan | | Kagoshima Pref. | TNS | 763119 | AB575248 | AB575859 | |  | |  |
|  | *Crypsinus hastatus* (Thunb.) Copel. | 1 | Japan | | Shizuoka Pref. | TNS | 763892 | AB575249 | AB575860 | |  | |  |
|  | *Crypsinus veitchii* (Baker) Copel. | 2 | Japan | | Nagano Pref. | TNS | 766630 | AB575250 | AB575861 | |  | |  |
|  | *Crypsinus yakuinsularis* (Masam.) Tagawa | 0 | Japan | | Kagoshima Pref. | TNS | 763337 | AB575251 | AB575862 | |  | |  |
|  | *Crypsinus yakushimensis* (Makino) Tagawa | 1 | Japan | | Kagoshima Pref. | TNS | 763136 | AB575252 | AB575863 | |  | |  |
|  | *Ctenopteris kanashiroi* (Hayata) K.Iwats.  (= *Prosaptia kanashiroi* (Hayata) Nakai ex Yamam.) | 0 | Japan | | Okinawa Pref. | TNS | 759271 | AB575253 | AB575864 | |  | |  |
|  | *Ctenopteris sakaguchiana* (Koidz.) H.Itô | 0 | Japan | | Nara Pref. | TNS | 776372 | AB575254 | AB575865 | |  | |  |
|  | *Drymotaenium miyoshianum* (Makino) Makino  (= *Lepisorus miyoshianus* (Makino) Fraser-Jenk. et Subh.Chandra) | 1 | Japan | | Hiroshima Pref. | TNS | 766504 | AB575255 | AB575866 | |  | |  |
|  | *Drynaria roosii* Nakaike | 0 | Japan | | Okinawa Pref. | TNS | 770530 | AB575256 | AB575867 | |  | |  |
|  | *Grammitis dorsipila* (H.Christ) C.Chr. et Tardieu | 0 | Japan | | Kagoshima Pref. | TNS | 764128 | AB575257 | AB575868 | |  | |  |
|  | *Grammitis tuyamae* H.Ohba | 0 | Japan | | Tokyo Pref. | MAK | Takayama 07062194 | AB575258 | AB575869 | |  | |  |
|  | *Lemmaphyllum microphyllum* C.Presl var. *microphyllum* | 1 | Japan | | Kagoshima Pref. | TNS | 763135 | AB575259 | AB575870 | |  | |  |
|  | *Lemmaphyllum microphyllum* C.Presl var. *obovatum* (Harr.) C.Chr. | 1 | Japan | | Okinawa Pref. | TNS | 759255 | AB575260 | AB575871 | |  | |  |
|  | *Lemmaphyllum pyriforme* (Ching) Ching | 0 | Japan | | Kagoshima Pref. | TNS | 766458 | AB575261 | AB575872 | |  | |  |
|  | *Lepisorus angustatus* Ching | 2 | Japan | | Saitama Pref. | TNS | 776409 | AB575262 | AB575873 | |  | |  |
|  | *Lepisorus annuifrons* (Makino) Ching | 1 | Japan | | Yamanashi Pref. | TNS | 776393 | AB575263 | AB575874 | |  | |  |
|  | *Lepisorus boninensis* (H.Christ) Ching | 1 | Japan | | Tokyo Pref. | TNS | 763872 | AB575264 | AB575875 | |  | |  |
|  | *Lepisorus clathratus* (C.B.Clarke) Ching | 2 | Japan | | Nagano Pref. | TNS | 776476 | AB575265 | AB575876 | |  | |  |
|  | *Lepisorus hachijoensis* Sa.Kurata | 1 | Japan | | Tokyo Pref. | TNS | 763996 | AB575266 | AB575877 | |  | |  |
|  | *Lepisorus oligolepidus* (Baker) Ching | 2 | Japan | | Nagano Pref. | TNS | 776486 | AB575267 | AB575878 | |  | |  |
|  | *Lepisorus onoei* (Franch. et Sav.) Ching | 1 | Japan | | Kagoshima Pref. | TNS | 762569 | AB575268 | AB575879 | |  | |  |
|  | *Lepisorus thunbergianus* (Kaulf.) Ching | 2 | Japan | | Miyazaki Pref. | TNS | 762752 | AB575269 | AB575880 | |  | |  |
|  | *Lepisorus tosaensis* (Makino) H.Itô | 2 | Japan | | Miyazaki Pref. | TNS | 762718 | AB575270 | AB575881 | |  | |  |
|  | *Lepisorus uchiyamae* (Makino) H.Itô | 2 | Japan | | Mie Pref. | TNS | 766444 | AB575271 | AB575882 | |  | |  |
|  | *Lepisorus ussuriensis* (Regel et Maack) Ching var. *distans* (Makino) Tagawa | 1 | Japan | | Kagoshima Pref. | TNS | 762572 | AB575272 | AB575883 | |  | |  |
|  | *Leptochilus decurrens* Blume  (= *Paraleptochilus decurrens* (Blume) Copel.) | 2 | Japan | | Tokyo Pref. | TNS | 766500 | AB575273 | AB575884 | |  | |  |
|  | *Loxogramme duclouxii* H.Christ | 2 | Japan | | Saitama Pref. | TNS | 1108348 | AB575274 | AB575885 | |  | |  |
|  | *Loxogramme grammitoides* (Baker) C.Chr. | 0 | Japan | | Nara Pref. | TNS | 766474 | AB575275 | AB575886 | |  | |  |
|  | *Loxogramme salicifolia* (Makino) Makino | 1 | Japan | | Kagoshima Pref. | TNS | 763133 | AB575276 | AB575887 | |  | |  |
|  | *Microsorum buergerianum* (Miq.) Ching | 2 | Japan | | Okinawa Pref. | TNS | 759349 | AB575277 | AB575888 | |  | |  |
|  | *Microsorum dilatatum* (Bedd.) Sledge | 1 | Japan | | Kagoshima Pref. | TNS | 763056 | AB575278 | AB575889 | |  | |  |
|  | *Microsorum fortunei* (T.Moore) Ching | 1 | Japan | | Tokyo Pref. | TNS | 774839 | AB575279 | AB575890 | |  | |  |
|  | *Microsorum rubidum* (Kunze) Copel. | 0 | Japan | | Okinawa Pref. | TNS | 759256 | AB575280 | AB575891 | |  | |  |
|  | *Microsorum scolopendria* (Burm.f.) Copel.  (= *Phymatosorus scolopendria* (Burm.f.) Pic.Serm.) | 1 | Japan | | Okinawa Pref. | TNS | 764387 | AB575281 | AB575892 | |  | |  |
|  | *Neocheiropteris ensata* (Thunb.) Ching var. *ensata* | 2 | Japan | | Kagoshima Pref. | TNS | 762545 | AB575282 | AB575893 | |  | |  |
|  | *Neocheiropteris ensata* (Thunb.) Ching var. *izuensis* Sa.Kurata et K.Satake | 0 | Japan | | Shizuoka Pref. | TNS | 1107849 | AB575283 | N/A | |  | |  |
|  | *Neocheiropteris subhastata* (Baker) Tagawa | 3 | Japan | | Kumamoto Pref. | TNS | 762683 | AB575284 | AB575894 | |  | |  |
|  | *Pleurosoriopsis makinoi* (Maxim. ex Makino) Fomin | 2 | Japan | | Akita Pref. | TNS | 765249 | AB575285 | AB575895 | |  | |  |
|  | *Polypodium amamianum* Tagawa | 1 | Japan | | Kagoshima Pref. | TNS | 764212 | AB575286 | AB575896 | |  | |  |
|  | *Polypodium fauriei* H.Christ | 1 | Japan | | Akita Pref. | TNS | 765157 | AB575287 | AB575897 | |  | |  |
|  | *Polypodium formosanum* Baker | 1 | Japan | | Kagoshima Pref. | TNS | 763316 | AB575288 | AB575898 | |  | |  |
|  | *Polypodium niponicum* Mett. | 1 | Japan | | Kumamoto Pref. | TNS | 762667 | AB575289 | AB575899 | |  | |  |
|  | *Polypodium someyae* Yatabe var. *someyae* | 1 | Japan | | Saitama Pref. | TNS | 774840 | AB575290 | AB575900 | |  | |  |
|  | *Polypodium someyae* Yatabe var. *awaense* Tagawa | 0 | Japan | | Kochi Pref. | TNS | 766624 | AB575291 | AB575901 | |  | |  |
|  | *Polypodium virginianum* L. | 1 | Japan | | Akita Pref. | TNS | 765156 | AB575292 | AB575902 | |  | |  |
|  | *Polypodium vulgare* L. | 2 | Locality uncertain | |  | TNS | 766503 | AB575293 | AB575903 | | | |  |
|  | *Pyrrosia adnascens* (Sw.) Ching | 0 | Taiwan | |  | TNS | 764058 | AB575294 | AB575904 | |  | |  |
|  | *Pyrrosia davidii* (Giesenh.) Ching | 0 | Japan | | Saitama Pref. | TNS | 774842 | AB575295 | AB575905 | |  | |  |
|  | *Pyrrosia hastata* (Houtt.) Ching | 1 | Japan | | Shizuoka Pref. | TNS | 763873 | AB575296 | AB575906 | |  | |  |
|  | *Pyrrosia linearifolia* (Hook.) Ching var. *linearifolia* | 1 | Japan | | Ehime Pref. | TNS | 763877 | AB575297 | AB575907 | |  | |  |
|  | *Pyrrosia linearifolia* (Hook.) Ching var. *heterolepis* Tagawa | 0 | Japan | | Okinawa Pref. | TNS | 736789 | AB575298 | AB575908 | |  | |  |
|  | *Pyrrosia lingua* (Thunb.) Farw. | 1 | Japan | | Kagoshima Pref. | TNS | 763117 | AB575299 | AB575909 | |  | |  |
|  | *Xiphopteris okuboi* (Yatabe) Copel.  (= *Micropolypodium okuboi* (Yatabe) Hayata) | 0 | Japan | | Miyazaki Pref. | TNS | 762780 | AB575300 | AB575910 | |  | |  |

Reproductive mode/Ploidy: 0=unknown, 1=sexual diploid, 2=sexual polyploid, 3=apogamous or sexual + apogamous. Information is based on Takamiya (1996) [45] and M. Takamiya (personal communication).
