# Supplementary material for: Molecular Species Identification with Rich Floristic Sampling: DNA Barcoding the Pteridophyte Flora of Japan
Source: PLoS One. 2010 Dec 8;5(12):e15136. doi: 10.1371/journal.pone.0015136 (PMC2999545; doi:10.1371/journal.pone.0015136)
Supplement: Table S2 — List of samples removed from the BLAST test. (DOC) [file pone.0015136.s002.doc]

Table S2 List of samples removed from the BLAST test.

*rbcL*:

*Isoetes asiatica*

*Pteris formosana*

*Thelypteris torresiana* var. *torresiana*

*trnH-psbA*:

*Arachniodes simplicior* var. *simplicior*

*Cheilanthes krameri*

*Cornopteris banajaoensis*

*Cornopteris crenulatoserrulata*

*Diplazium kawakamii*

*Diplazium mettenianum*

*Dryopteris fuscipes*

*Dryopteris gymnosora*

*Dryopteris hasseltii*

*Dryopteris simasakii* var. *simasakii*

*Polystichum piceopaleaceum*
